# Environmental enteric dysfunction pathways and child stunting: A systematic review

**DOI:** 10.1371/journal.pntd.0006205

**Published:** 2018-01-19

**Authors:** Kaitlyn M. Harper, Maxine Mutasa, Andrew J. Prendergast, Jean Humphrey, Amee R. Manges

**Affiliations:** 1 School of Population and Public Health, University of British Columbia, Vancouver, Canada; 2 Faculty of Health Sciences, Simon Fraser University, Burnaby, Canada; 3 Blizard Institute, Queen Mary University of London, London, United Kingdom; 4 Zvitambo Institute for Maternal and Child Health Research, Harare, Zimbabwe; 5 Department of International Health, Johns Hopkins Bloomberg School of Public Health, Baltimore, Maryland, United States of America; 6 British Columbia Centre for Disease Control, Vancouver, Canada; The Johns Hopkins University, UNITED STATES

## Abstract

**Background:**

Environmental enteric dysfunction (EED) is commonly defined as an acquired subclinical disorder of the small intestine, characterized by villous atrophy and crypt hyperplasia. EED has been proposed to underlie stunted growth among children in developing countries. A collection of biomarkers, organized into distinct domains, has been used to measure different aspects of EED. Here, we examine whether these hypothesized relationships, among EED domains and between each domain and stunting, are supported by data from recent studies.

**Methodology:**

A systematic literature search was conducted using PubMed, MEDLINE, EMBASE, Web of Science, and CINAHL between January 1, 2010 and April 20, 2017. Information on study objective, design, population, location, biomarkers, and results were recorded, as well as qualitative and quantitative definitions of EED. Biomarkers were organized into five EED domains, and the number of studies that support or do not support relationships among domains and between each domain with stunting were summarized.

**Results:**

There was little evidence to support the pathway from intestinal permeability to microbial translocation and from microbial translocation to stunting, but stronger support existed for the link between intestinal inflammation and systemic inflammation and for intestinal inflammation and stunting. There was conflicting evidence for the pathways from intestinal damage to intestinal permeability and intestinal damage to stunting.

**Conclusions:**

These results suggest that certain EED biomarkers may require reconsideration, particularly those most difficult to measure, such as microbial translocation and intestinal permeability. We discuss several issues with currently used biomarkers and recommend further analysis of pathogen-induced changes to the intestinal microbiota as a pathway leading to stunting.

## Author summary

Globally, one-quarter of children under the age of five are affected by poor linear growth, known as stunting. Interventions, including giving children supplemental foods or improving hygiene to prevent diarrhea, have only been partially successful at restoring normal growth. Environmental enteric dysfunction (EED) is a disease characterized by damage to the lining of the small intestine and is thought to contribute to stunting, though the exact mechanism is still unclear. EED was first diagnosed by removing samples of the intestinal lining and analyzing them under a microscope; however, these procedures are costly and invasive, and recent research has focused on discovering easier ways to identify EED. These tests focus on the many interconnected aspects of EED, including damage and function of the intestinal wall, inflammation, and presence of pathogenic bacteria outside the gut. We conducted a systematic review to evaluate the evidence of relationships between each aspect of EED and stunting. We found the most evidence for the relationship between inflammation and stunting, but less evidence for the relationship between stunting and the presence of pathogenic bacteria outside the gut. Our results suggest that EED may be more complex than previously conceived and that some frequently used EED tests may need reconsideration.

## Introduction

One-quarter of children under the age of 5 years are stunted, defined as a height-for-age > 2 standard deviations below the median as defined by the World Health Organization growth standards. Children whose linear growth is impaired during the first 1000 days after conception have an increased risk of poor cognitive development and educational performance, lost productivity and lower adult earnings, chronic diseases, and mortality over their lifetime [1–3].

There is a well-recognized network of interacting determinants that underlie stunting [4–13]. For many years, studies have focused predominantly on nutrition-specific interventions for stunting; however, previous systematic reviews highlight that neither food quantity nor quality fully explains impaired linear growth in children [14,15]. Diarrhea has been proposed as a major contributor to growth failure in young children, though results are inconsistent [16–19]. While diarrheal episodes in the first few months after birth lead to increased prevalence of stunting at 24 months [20], catch-up growth between diarrheal episodes can be sufficient for linear growth recovery in some children [21].

Environmental enteric dysfunction (EED) is commonly defined as an acquired subclinical disorder of the small intestine, characterized by villous atrophy and crypt hyperplasia. Previous reviews have described the history and epidemiology of environmental enteric dysfunction in detail [1,22–26] and have refocused attention on EED as a potential cause of stunting in developing countries. Exposure to bacteria through fecal contamination is postulated to induce morphological changes, leading to intestinal epithelial damage, increased permeability, and microbial translocation into the lamina propria. This invasion prompts an influx of inflammatory cells to the intestine and leads to local and systematic inflammation, resulting in the reallocation of resources normally directed toward child growth and development, and disruption of hormonal pathways that regulate growth plate activity in long bones. Chronic inflammation and reduced intestinal nutrient absorption are also hypothesized to affect brain development, inducing lasting negative effects on cognition, educational achievement, and linear growth [27].

There are currently no clear diagnostic criteria for EED, which presents a major problem in investigating the role of EED in stunting, and in evaluating treatment and prevention strategies. Intestinal biopsy is used to diagnose diseases with similar pathological changes, such as celiac disease [28]; however, collection of small bowel biopsy samples is technically and ethically infeasible in young children. Over recent years, studies have evaluated a range of potential biomarkers of EED, with a general agreement that these should be organized into distinct domains to measure different aspects of the pathogenic pathway that characterizes EED. Studies have included noninvasive biomarkers of intestinal damage and repair, epithelial permeability and absorption, digestion, epithelial morphology, intestinal inflammation, microbial drivers, systemic immune activation, and non-small intestine organ function.

Multiple research groups have used this domain-based approach, focusing on longstanding physiologic relationships to study the complex mechanisms that may underlie EED. Here, we examine whether these relationships are supported by recent and rich new data from studies conducted between 2010–2017, building on the review conducted by Denno et al for the time period 2000–2010 [29]. We define five contributing domains of EED to provide supporting evidence for our two aims: (i) to evaluate the relationships between individual EED domains; and (ii) to evaluate the relationships between each EED domain and stunting. We focus on stunting as the primary outcome in this review, as it is the most common outcome of the included studies and is objectively measured. Although our ultimate interest is in cognition and child development, these have not been as commonly measured, and the mechanistic pathways between stunting and neurodevelopment remain unclear [30].

## Methods

### Search strategy and selection criteria

Our search strategy followed PRISMA (Preferred Reporting Items for Systematic Reviews and Meta-Analyses) guidelines for the reporting of systematic reviews [31]. A search for articles in any language between January 1, 2010, and April 20, 2017, was conducted using PubMed, MEDLINE, EMBASE, Web of Science, and CINAHL. Abstracts were independently screened by two reviewers (K.H. and M.M.) and full-text articles that were related in any way to “environmental enteric dysfunction”, “environmental enteropathy”, or “tropical enteropathy” were selected for review. The reference lists of all review articles and original publications were also screened for any relevant studies. Published study abstracts were also included in this review. Disagreements regarding study inclusion were resolved by consensus.

### Characteristics of study populations

Our population of interest included individuals of all ages for whom two or more EED domains, or at least one domain and stunting, were measured. Only human studies were included. While most studies focused on children under 5 years, no age restriction was imposed in the search criteria as some adult studies provide valuable histopathological data that is less frequently collected in young children. Studies in both developed and developing countries were included in the search, though only one study in the final selection included individuals from a developed country [32].

### Data abstraction

Studies selected for inclusion can be categorized into three groups: (i) observational studies in which EED was defined as either an exposure or as an outcome; (ii) studies investigating potential EED biomarkers and/or identification/diagnosis of EED; and (iii) intervention studies designed to treat or prevent EED. A standardized data abstraction form was used to extract information from each study. Abstracted data included: study objective, study design, location, subject eligibility and description of study population, inclusion or exclusion of subjects with diarrhea or human immunodeficiency virus (HIV), results and final conclusions. Biomarkers and diagnostic tests were recorded, as well as any qualitative and quantitative definitions of EED provided by the authors (S1 Table).

### Definition of EED domains

To review the evidence supporting the complex mechanisms that may contribute to child stunting we have elected to organize results according to these five EED domains: (1) intestinal damage and repair, (2) permeability and absorption, (3) microbial translocation, (4) intestinal inflammation, and (5) systemic inflammation. These domains were determined by consensus from reviewing previous conceptual frameworks and descriptions [2,3,23,24,29,33–41]. Each domain and its respective non-invasive biomarkers are described below.

#### Markers of intestinal damage and repair

Citrulline is a non-essential amino acid mainly produced by enterocytes *de novo*. Circulating concentrations of citrulline therefore reflect total enterocyte mass and low concentrations indicate reduced surface area [42]. Intestinal fatty acid binding protein (I-FABP) is an intracellular epithelial protein located primarily at the tips of small intestinal villi that is rapidly released into the circulation after injury to the epithelia. It has been used to indicate the severity of intestinal damage in both adults [43] and children [44] and has a very short half-life, reflecting recent intestinal injury. Regenerating (REG) family proteins are involved in tissue regeneration and cell proliferation [45,46], and increased concentrations of fecal REG proteins indicate epithelial injury [47,48]. Glucagon-like peptide 2 (GLP-2) is a gut trophic factor released by enteroendocrine L-cells of the ileum. GLP-2 aids in mucosal regeneration, leading to increased villus length, improved gut barrier and absorptive functions, and anti-inflammatory mucosal activity [49].

#### Markers of permeability and absorption

The most widely accepted biomarker for epithelial integrity and permeability is the dual-sugar absorption test, although it has several limitations [50]. Intestinal inflammation is thought to create small pores between epithelial cells allowing for paracellular permeation of lactulose, while villous atrophy reduces epithelial surface area and mannitol (or rhamnose) absorption. A higher lactulose-mannitol (L:M) or lactulose-rhamnose (L:R) ratio has been used as an indication of EED, although recovery of the two sugars is sometimes reported separately to disaggregate absorption and permeability. However, because many studies report these measurements only as a ratio, we chose to combine these measurements into one single domain.

Alpha-1-Antitrypsin (AAT) is a protein released during inflammation to protect cells against proteolytic enzymes released by neutrophils during infection [51]. However, AAT is not synthesized in the gut, and the presence of AAT in stool reflects protein loss and increased permeability from the blood into the gut lumen. Claudins and zonulin are proteins that modulate tight junctions, which form paracellular barriers between intestinal epithelial cells, thereby governing permeability and selectivity. High concentrations of zonulin suggest increased permeability [52]. Increased concentrations of claudin-2 and -15 indicate decreased intestinal absorption; increased concentration of claudin-4 indicates increased cell shedding [53].

#### Markers of microbial translocation

Microbial translocation is defined as the passage of microbes or microbial products, which are strongly immunogenic, through the epithelial barrier into the lamina propria and local mesenteric lymph nodes [54]. Lipopolysaccharide (LPS) and flagellin, two outer components of bacterial structure, are frequently used to indicate microbial translocation. Elevated plasma endotoxin core antibody (EndoCAb) titers and anti-LPS immunoglobulin G (IgG) and A (IgA) are used to identify an immune response to systemic LPS [55–57].

#### Markers of intestinal inflammation

Translocated LPS and other microbial products engage the mucosal immune system, prompting the activation of inflammatory cells such as neutrophils, macrophages and dendritic cells. Myeloperoxidase (MPO), an enzyme stored inside neutrophils, is involved in the process of killing bacteria [58]. Neopterin (NEO) is produced by macrophages or dendritic cells upon stimulation by interferon-gamma [59], which is released during pro-inflammatory responses by Th1 lymphocytes [60]. Additionally, it is hypothesized that a combination of biomarkers may explain linear growth deficits better than any single biomarker[61], and some studies calculate an “EED composite score” comprised of three fecal biomarkers—AAT, MPO, and NEO [41,61–63]. While AAT is primarily a measure of intestinal permeability, we include the measures of the composite score in the intestinal inflammation domain as each biomarker may be used to measure aspects of gut inflammation.

Calprotectin is a calcium- and zinc-binding protein released by neutrophils as a result of cell stress or damage and is excreted in feces [64]. Accordingly, fecal calprotectin has been used as an indicator of gut damage [65]. However, in young healthy breastfed infants, fecal calprotectin concentrations are high [66] and decline with increasing infant age [67]. Therefore, high fecal calprotectin levels in young infants appears to be physiologic rather than an indicator of gut inflammation.

#### Markers of systemic inflammation

Interferon gamma (IFN-γ), tumour necrosis factor (TNF), and interleukins (e.g., IL-6, IL-10) are cell-signalling cytokines that activate and drive differentiation of immune cells upon infection [68]. Alpha-1-acid glycoprotein, C-reactive protein, and ferritin are upregulated acute-phase proteins involved in the inhibition of microbial growth. Soluble CD14 (sCD14) is a circulating co-receptor for LPS secreted by monocytes and macrophages [69]. Other systemic markers include total IgG and IgM, which are released by antibody-secreting B-lymphocytes, termed plasma cells. The kynurenine-tryptophan ratio (KTR) also indicates immune activation [70]: kynurenine is formed from the essential amino acid tryptophan by the enzyme indolamine 2,3-dioxygenase, which is upregulated by pro-inflammatory cytokines such as IFN-γ. Immune activation therefore leads to formation of kynurenine and depletion of tryptophan, and a higher KTR indicates a systemic immune response.

### Study inclusion and synthesis of results

Studies were included if they reported results for two or more EED domains, or at least one domain and stunting. In the situation where multiple studies included the same EED measurements on the same study subjects, only the most complete report was included in the review. For each of the 5 EED domains, we first present the number of studies that support (or not) the relationships of each domain to stunting. Second, we present the number of studies that report data in support of the relationship between each EED domain and the other domains, where data are available. These associations are summarized in Tables 1 and 2.

**Table 1:** Evidence of association between EED domains.

Evidence that supports pathway	Evidence that does not support pathway
**Intestinal Damage and Repair—Permeability and Absorption**	
**2017 Semba:** L:M associated with low citrulline and high homocitrulline	**2013 Agapova:** REG1B was not significantly different between children with high and low L:M
**2013 Agapova:** REG4 gene best differentiated children with increased L:M from children with normal L:M (p = 0.01)	**2016 Kosek:** Citrulline not associated with %M
**2016 Ordiz:** REG1A gene was part of a model that predicted severe EED (LM > 0.45)	**2015 Gosselin:** No association between L:M and citrulline
**2016 Guerrant:** REG1B associated with AAT	**2013 Wessells:** Citrulline not associated with L:M recovery ratio
**2016 Kosek:** %L negatively associated with citrulline	**2010 Papadia:** Citrulline not associated with rhamnose:glucose ratio or lactulose:rhamnose ratio after Benjamini-Hochberg correction
**2010 Papadia:** Citrulline associated with xylose absorption in HIV-positive individuals after Benjamini-Hochberg correction	
**Intestinal Damage and Repair—Systemic Inflammation**	
**2016 Kosek:** Citrulline inversely correlated with CRP, AGP, IL-6 at 7,15,24 months	
**2016 Guerrant:** Citrulline negatively correlated with KTR	
**Intestinal Damage and Repair—Intestinal Inflammation**	
**2016 Guerrant:** REG1B associated with MPO and NEO	
**Intestinal Damage and Repair—Microbial Translocation**	
**2016 Guerrant:** REG1B associated with anti-LPS antibodies	
**2016 Kelly:** Log-transformed LPS associated with cell shedding; LPS associated with epithelial perimeter and villous surface area-volume ratio; LPS inversely associated with GLP2	
**2016 Uddin:** I-FABP positively associated with anti-LPS IgG	
**2016 Campbell:** GLP-2 inversely associated with EndoCAb	
**Permeability and Absorption—Intestinal inflammation**	
**2017 Campbell:** AAT was modestly correlated with MPO (r = 0.33, p<0.01)	**2017 Kosek:** No biomarkers of intestinal inflammation associated with L:M z-score at age 2
**2017 Kosek:** MPO associated with L:M z-score at age 1	**2017 Campbell**: No significant correlation of L:M with any biomarkers of intestinal inflammation
**2016 Guerrant:** MPO associated with both L:M and %L; zonulin negatively associated with MPO and NEO; Claudin-15 negatively associated with L:M and AAT	**2013 Agapova:** Transcripts for MPO and calprotectin were not significantly different between children with high and low L:M
**2015 George:** Calprotectin associated with MPO; AAT associated with MPO	
**Permeability and Absorption—Systemic Inflammation**	
**2016 Ordiz:** TNF, CD53 associated with L:M (sensitivity = 84%, specificity = 83%)	**2013 Agapova:** Transcripts of TNF, IFN-γ, and other markers of systemic inflammation were not significantly different between children with high and low L:M
**2016 Yu**: 51 fecal transcripts involved in systemic inflammation associated with %L	**2013 Wessells:** AGP and CRP not associated with L:M
**2016 Semba**: KTR significantly correlated with gut permeability; serotonin:tryptophan ratio also correlated with gut permeability	
**Permeability and absorption—Microbial translocation**	
**2016 Guerrant:** Zonulin associated with anti-LPS IgG and anti-FliC IgG; L:M weakly associated with anti-LPS IgG	**2017 Campbell:** No significant correlation of L:M with other biomarkers
**2010 Kelly:** Log-transformed xylose recovery negatively correlated with log-transformed anti-LPS IgG (p = 0.006)	**2016 Guerrant:** L:M not significantly associated with LPS, anti-LPS IgA, anti-FliC IgG/IgA; %L not significantly associated with LPS, anti-LPS IgA/ IgG, anti-FliC IgG/IgA
	**2015 Benzoni:** No significant association between log-EndoCAb and log-%L or log-L:M
	**2010 Kelly:** No associations between anti-LPS antibodies or LPS and %R, %L, or glucose
**Microbial translocation—Systemic inflammation**	
**2017 Campbell:** EndoCAb and total IgG/IgM were consistently intercorrelated	**2016 Kelly:** Plasma LPS was not associated with CRP, sCD14, CD163, or LPS-binding protein
**2016 Uddin:** anti-LPS antibodies positively associated with sCD14 (p = 0.07)	
**2010 Kelly:** Correlation between TNF receptor p55 and anti-LPS IgG and IgM	
**Microbial translocation—Intestinal inflammation**	
**2016 Uddin:** MPO positively associated with anti-LPS antibodies	
**Intestinal inflammation—Systemic inflammation**	
**2017 Kosek:** At age 2, MPO and AGP were positively correlated; NEO negatively correlated with AGP	**2017 Campbell:** Systematic inflammation loaded on their own components when added to the PCA model rather than combining with gut markers
**2016 Guerrant:** KTR and CRP associated with MPO; KTR associated with CRP	
**2015 Naylor:** Inclusion of both intestinal and systemic inflammatory markers in clusters 1 and 2: (i) systemic inflammatory markers plus diarrheal burden; (ii) intestinal inflammatory markers plus CRP and sCD14	

Evidence supporting or not supporting the pathways between five domains of EED. Empty cells indicate that no included studies contained evidence to support or not support the pathway between domains. Abbreviations: AAT, alpha-1 antitrypsin; AGP, alpha-1-acid glycoprotein; CRP, C-reactive protein; EndoCAb, endotoxin-core antibody; GLP-2, glucagon-like peptide-2; I-FABP, intestinal fatty acid-binding protein; IFN-γ, interferon gamma; IGF, insulin-like growth factor; IgG, immunoglobulin G; IgM, immunoglobulin M; KTR, kynurenine-tryptophan ratio; L:M, lactulose-mannitol ratio; LPS, lipopolysaccharide; L:R, lactulose-rhamnose ratio; MPO, myeloperoxidase; NEO, neopterin; REG, regenerating islet derived protein; sCD14, soluble CD14; TNF, tissue necrosis factor; %L, percent lactulose permeability; %M, percent mannitol absorption.

**Table 2:** Evidence of association between EED domains and stunting.

Evidence that supports pathway	Evidence that does not support pathway
**Intestinal Damage and Repair—Growth**	
**2016 Guerrant:** HAZ associated with lower citrulline at baseline**;** Higher citrulline predicted less stunting in girls only**;** Lower HAZ associated with higher I-FABP at baseline; Higher I-FABP associated with ΔHAZ	**2016 Kosek:** Tanzania site only: No association between citrulline and HAZ
**2016 Kosek:** In Peru, lower levels of citrulline at 3 mo were associated with higher stunting over subsequent 6 mo	**2015 Gosselin:** Malawi, no significant difference in mean citrulline levels between stunted and non-stunted children; Tanzania, citrulline did not predict subsequent stunting
**2013 Peterson:** Higher REG1B concentrations at 3 mo significantly associated with future HAZ in cohorts of Peruvian and Bengali children	**2014 Prendergast:** I-FABP not associated with stunting; I-FABP increased between 3–12 mo and decreased between 12–18 mo
	**2017 Campbell:** Higher GLP-2 associated with lower HAZ in children 6 mo of age
**Permeability and Absorption—Growth**	
**2017 Campbell:** Gut permeability score associated with growth at 6 mo and between 6–18 mo	**2017 Brown:** Children with higher HAZ (i.e. taller children) had higher L:C ratio
**2016 Ordiz:** L:M associated with ΔHAZ at 3 mo	**2017 Kosek:** HAZ not associated with L:M z-score
**2016 Guerrant:** ΔHAZ associated with higher L:M**;** Zonulin associated with stunting in children > 12 mo	**2017 Semba:** HAZ not associated with L:M
**2016 Faubion:** %L significantly predicted HAZ	**2017 Faubion:** HAZ not associated with %R nor L:R
**2016 Yu:** %L was associated with reduced ΔHAZ	**2017 Ordiz:** Baseline HAZ not associated with L:M
**2015 Lima:** Higher AAT associated with impaired catch-up growth	**2016 Arndt:** AAT was not associated with subsequent 3-month linear growth
**2013 Kosek:** AAT predicted declines in HAZ in subsequent 6 mo	**2013 Wessells:** HAZ not associated with L:M recovery ratio at baseline
**2013 Lin:** L:M strongly associated with HAZ	**2012 Weisz:** HAZ not associated with L:M
**2012 Weisz:** %L associated with ΔHAZ	
**Microbial translocation—Growth**	
**2016 Guerrant:** Lower HAZ associated with IgA anti-flagellin and anti-LPS antibodies at baseline; Higher LPS associated with smaller ΔHAZ	**2017 Campbell:** Higher EndoCAb associated with greater baseline HAZ
**2016 Syed:** Higher anti-LPS IgA levels were associated with a decrease in ΔHAZ from 6–18 months	**2016 McDonald:** Microbial translocation associated with underweight but not stunting**;** None of the biomarkers were significantly associated with risk of stunting in either the unadjusted or adjusted models
**2014 Jones:** EndoCAb at day 28 negatively correlated with growth at day 56; EndoCAb positively correlated with IGF-1 at day 56	**2016 Syed:** anti-LPS IgG not associated with ΔHAZ
	**2015 Benzoni:** No significant association between log-EndoCab and HAZ or ΔHAZ
	**2014 Prendergast:** No association between EndoCAb and stunting
	**2013 Lin:** HAZ not strongly associated with IgG EndoCAb
**Intestinal inflammation—Growth**	
**2017 Kosek:** Negative association between MPO and ΔHAZ at age 1	**2017 Campbell:** Gut inflammation score (MPO, NEO, AAT) was not associated with growth
**2016 Arndt:** High MPO levels were associated with decreases in 3-mo growth at age 2; Kosek Score barely associated with HAZ in mo 1–3 and mo 12–21	**2016 Arndt:** NEO was not associated with subsequent growth**;** MPO not associated with 6-mo linear growth at any time**;** Kosek Score not associated w/LAZ in mo 3–9 nor any other time when Benjamini-Hochberg adjustments were applied
**2016 Guerrant:** MPO associated with ΔHAZ	**2015 George:** Baseline markers of intestinal inflammation (calprotectin and EED activity score) were not associated with stunting
**2016 Naylor:** Calprotectin at 12 wks negatively correlated with HAZ**;** MPO associated with ΔHAZ from enrolment to age 1	
**2015 Lima:** MPO associated with impaired catch-up growth	
**2013 Kosek:** Kosek Score associated with decreased HAZ in subsequent 6 mo in Brazil, Nepal, and South Africa; NEO predicted declines in HAZ in subsequent 6 mo	
**2014 Jones:** IGF-1 weakly associated with calprotectin at day 56	
**Systemic inflammation—Growth**	
**2017 Kosek:** Higher AGP concentrations were associated with decreased ΔHAZ at both ages	**2016 Guerrant:** CRP not associated with stunting
**2016 Kosek:** KTR significantly inversely related to ΔHAZ in Tanzania	**2016 Kosek:** KTR not significantly associated with ΔHAZ in Peru
**2016 Naylor:** CRP, ferritin, sCD14 strongly negatively correlated with ΔHAZ	**2014 Prendergast:** No associations between levels of IL-6 or sCD14 and stunting
**2014 Prendergast:** Higher log_10_ levels of CRP and AGP between 6 wk–12 mo were associated with increased odds of stunting; At birth, IGF-1 was strongly associated with AGP, CRP, and sCD14	**2013 Lin:** Total IgG not strongly associated with HAZ
**2014 Jones:** CRP, IL-1, IL-10 significantly negatively associated with IGF-1 at baseline but not day 28 or day 56	**2014 Jones:** No association between IGF-1 and sCD14 nor TNF; IL-7, IL-8, IL-15, IL-17a, IL-22, IFN-γ not associated with IGF-1

Evidence supporting or not supporting the pathways between each of the five EED domains and stunting. Abbreviations: AAT, alpha-1 antitrypsin; AGP, alpha-1-acid glycoprotein; CRP, C-reactive protein; EndoCAb, endotoxin-core antibody; GLP-2, glucagon-like peptide-2; HAZ, height-for-age z-score; I-FABP, intestinal fatty acid-binding protein; IFN-γ, interferon gamma; IGF, insulin-like growth factor; IgG, immunoglobulin G; IgM, immunoglobulin M; KTR, kynurenine-tryptophan ratio; L:M, lactulose-mannitol ratio; LPS, lipopolysaccharide; L:R, lactulose-rhamnose ratio; mo, month(s); MPO, myeloperoxidase; NEO, neopterin; REG, regenerating islet derived protein; sCD14, soluble CD14; TNF, tissue necrosis factor; wk(s), week(s); Δ, change; %L, percent lactulose permeability; %M, percent mannitol absorption; %R, percent rhamnose absorption.

## Results

**Fig 1:**
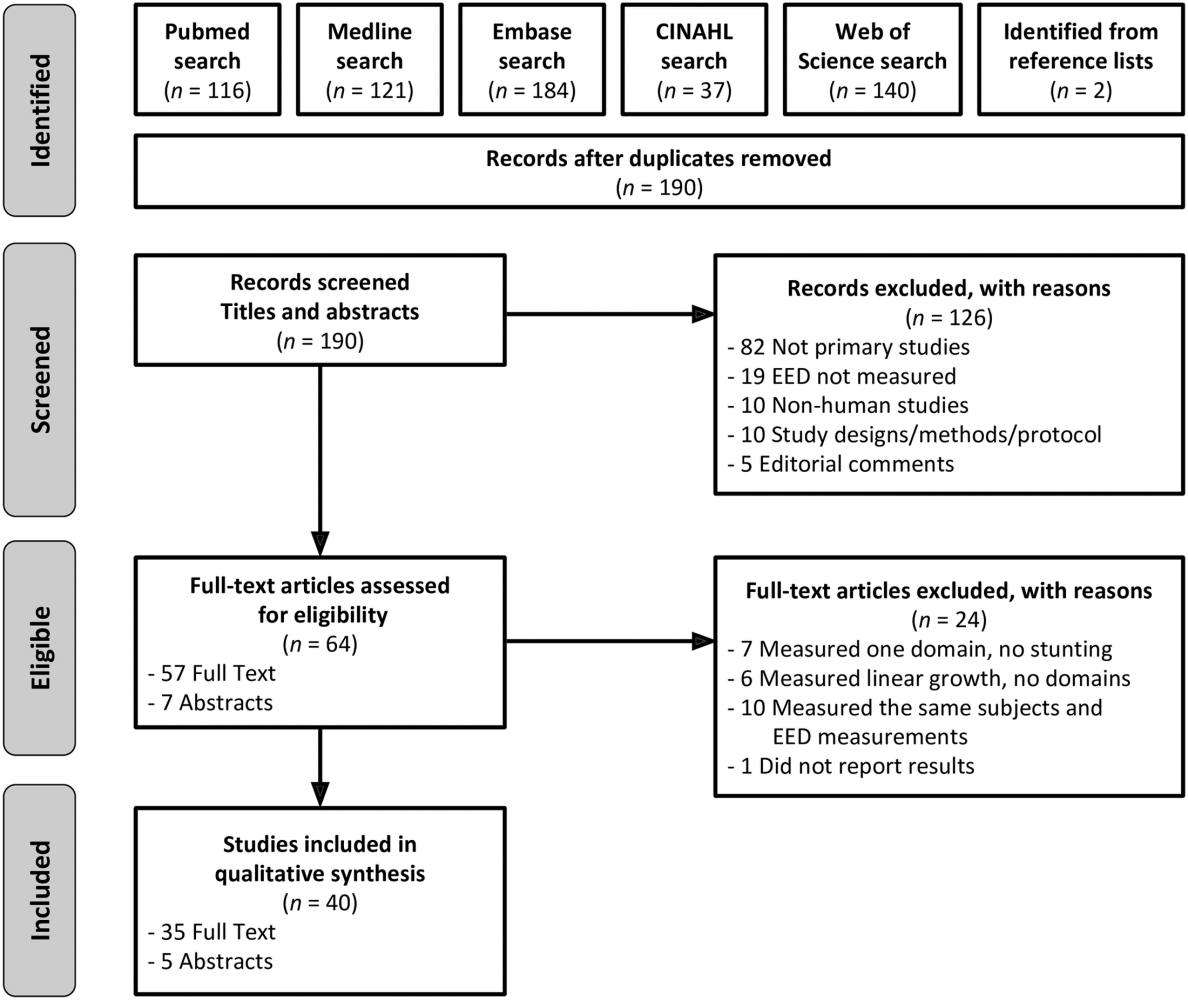
Flow diagram of literature search, review, and selection according to PRISMA.

The electronic search identified 598 potentially relevant abstracts and articles. Two additional records were identified from checking reference lists. A total of 190 records remained after duplicates were removed. 126 articles were excluded based on a review of the title and abstract (Fig 1). Of the 64 reports selected for full-text review, 24 were excluded: 7 studies reported results for one domain but did not include measurements of linear growth [71–77], 6 studies reported measurements of linear growth but did not report domain measurements [78–83], and 1 study abstract did not report results for domains or stunting [84]. Five abstracts [85–89] and five full-text articles [90–94] included overlapping EED measurements on the same study subjects and were excluded from our review. The final review therefore includes 40 reports (5 conference abstracts, 35 full articles).

### Study populations

Of the 40 reports included in this analysis, 10 (25%) of the studies were conducted in Central or South America, 16 (40%) were conducted in Asia, and 29 (73%) were conducted in Africa. Half of the studies were conducted in rural locations, and 1 study was conducted in both urban and rural populations, while 9 (23%) studies did not specify the setting. Thirty-six (90%) reports included participants under the age of 5 years, and 19 (48%) studies were restricted to children less than 2 years. Three studies were conducted exclusively in adults. Diarrhea was used as an exclusion criterion in 16 (40%) studies; 16 (40%) studies included participants regardless of diarrheal status and 8 (20%) studies did not mention diarrhea in their analyses. Twenty-nine (73%) reports did not specify the HIV status of participants. Seven (17%) studies used HIV as an exclusion criterion and 4 (10%) studies included individuals regardless of HIV status.

### Markers of intestinal damage and repair

#### Histopathology

Histopathological analysis of small bowel biopsies is used to assess intestinal mucosal damage. Four studies [53,75,77,95] included in this review evaluated severity of EED in Zambian adults, and therefore could not measure associations between intestinal morphology and child stunting. One study observed an association between histology of small intestinal biopsies and noninvasive markers of intestinal damage and repair [95] and a second study reported associations with microbial translocation [53].

#### Markers of intestinal damage and repair

I-FABP, REG1, GLP-2, and citrulline were used to investigate the possible associations between noninvasive markers of intestinal damage and repair with other domains and with stunting. Two studies observed significant associations between biomarkers of intestinal damage and repair and linear growth outcomes [96,97]. Two studies reported no association [98,99] and 1 study found conflicting results by study location [70]. One study reported an inverse relationship between GLP-2, an intestinotrophic factor, and linear growth [63]. GLP-2 has been associated with increased nutrient absorption in previous studies [100]; these results suggest that EED growth effects may not be improved by increased nutrient absorption alone.

Markers of intestinal damage and repair were not associated with the L:M [98,101] or L:R ratio [95] in 3 studies, and 2 studies found an inverse relationship between markers of intestinal damage and repair and the L:M test [102] or percent lactulose permeability (%L) [70]. A weak association with absorption was observed in HIV-positive adult individuals [95] but not in young children [70]. REG genes were significantly differentiated in children with increased L:M versus children with normal L:M in one study [103] and were associated with makers of intestinal permeability in two studies [96,104]. Markers of intestinal damage and repair were associated with markers of systemic inflammation in 2 studies [70,96] and with intestinal inflammation in a single study [96]. Intestinal damage and repair was associated with microbial translocation in 4 studies [53,96,63,105], but was not reported in 1 study [106]. In summary, the association between biomarkers of intestinal damage and repair with other domains and with stunting were largely conflicting, but evidence consistently supported the pathway between intestinal damage and repair and microbial translocation.

#### Markers of permeability and absorption

The dual-sugar test, often expressed as a ratio of lactulose and mannitol (L:M) or lactulose and rhamnose (L:R), is a combined measure of permeability and absorption expected to be inversely associated with linear growth. AAT, zonulin, claudin-4, and claudin-15 are other biomarkers used to measure permeability and absorption. Four studies observed a significant association between permeability/absorption and linear growth [96,63,104,107] and three additional studies found associations between permeability only (%L) and linear growth [32,108,109]. Higher levels of AAT were associated with impaired catch-up growth in one study [110] and predicted declines in HAZ for six months following the test [61].

Five studies observed no association between the permeability/absorption ratio and linear growth [101,108,111–113]. Moreover, AAT was not associated with subsequent 3-month linear growth [41]. One study abstract reported an unexpected positive relationship between linear growth and dual-sugar absorption [114]. However, this study used lactose and creatinine as tracers, which may not accurately measure permeability and absorption. Three studies did not report the relationship of linear growth with permeability/absorption [115–117]. In summary, evidence supporting the pathway between permeability/absorption and linear growth was split almost evenly across recent studies.

Increased permeability is hypothesized to be associated with microbial translocation and intestinal inflammation. Permeability may enable translocation into the lamina propria and cause intestinal inflammation; conversely, intestinal inflammation may open up tight junctions to increase permeability and microbial translocation [22,118,119]. One study observed a weak association of anti-LPS IgG with the dual-sugar test; however, measurements of LPS, anti-LPS IgA, anti-FliC IgG, and anti-FliC IgA were not associated with the dual-sugar test or with %L alone in the same study [96]. No association between microbial translocation and permeability/absorption was observed in 2 studies [55,63], and 1 study observed conflicting results between the two domains [120]. Two studies did not report direct associations between permeability/absorption and microbial translocation [33,107].

Intestinal inflammation was associated with permeability/absorption in three studies [96,113,62]**.** In one study, MPO—a marker of intestinal inflammation—was modestly correlated with AAT, but there were no significant correlation between any markers of intestinal inflammation and the dual-sugar test [63]. In a large multi-site study, MPO was associated with L:M z-score (standardized by age and sex) at age 1, but no intestinal inflammation biomarkers were associated with L:M z-score at age 2 [113].

Systemic inflammation was associated with permeability in a study of children in rural Malawi [102] but not in Burkina Faso [101]**.** Fecal transcriptomics analysis identified eighteen host mRNA transcripts associated with permeability/absorption, 7 of which were identified as predictors of EED with 84% sensitivity and 73–83% specificity, in a population of rural Malawian children [104]. A study of the same population found 51 transcripts correlated with permeability alone, almost all of which coded for proteins that regulate the systemic immune system [109]. However, a third study in the same geographic location found no association of the dual-sugar test with markers of systemic inflammation or intestinal inflammation [103]. In summary, the dual-sugar test was inconsistently associated with intestinal inflammation, systemic inflammation, and microbial translocation.

#### Markers of microbial translocation

The presence of anti-lipopolysaccharide (LPS) and anti-flagellin antibodies is used to indicate the host response to microbial translocation. Two studies observed an association of microbial translocation with linear growth [96,121]. In contrast, 4 studies found no association between microbial translocation and linear growth [55,99,107,122], and one study reported an unexpected positive association between microbial translocation and linear growth [63]. One study observed conflicting results between microbial translocation and linear growth [123], and 3 studies did not report associations [33,53,105].

Of the 7 studies that examined microbial translocation and inflammation, only 4 reported relationships; one study found positive associations of anti-LPS antibodies with markers of both systemic inflammation and intestinal inflammation [105]. Two studies observed associations of microbial translocation with markers of systemic inflammation [63,120], while another study found no association [53]. The remaining 3 studies did not report associations between microbial translocation and inflammation [33,105,121]. In summary, the pathway between microbial translocation and linear growth was not well supported, and there was inconsistent support for the pathways between microbial translocation and other domains.

#### Markers of intestinal inflammation and linear growth

Intestinal inflammation is a key feature of EED, and several fecal markers—MPO, NEO, and calprotectin—indicate intestinal inflammation. Individual biomarkers of intestinal inflammation were associated with or significantly predicted linear growth in 4 studies [61,96,113,124] and were associated with catch-up linear growth in 1 study [110]. Moreover, one study found a weak association between a marker of intestinal inflammation and insulin-like growth factor 1, a mediator of growth hormone that is important in childhood growth and development [121]. One study found no association of linear growth with baseline markers of intestinal inflammation [62], and one found conflicting results between growth and different intestinal biomarkers [41]**.** Overall, there was strong evidence supporting the pathway between intestinal inflammation and linear growth.

The EED composite score—comprised of MPO, NEO, and AAT—accurately predicted change in linear growth in a study of infants [61]. However, the baseline composite score was not associated with stunting [62] or HAZ at 18 months [63]. In one study, the unadjusted composite score was mildly associated with HAZ in months 1–3 and 12–21, but associations were not observed after adjustments were applied [41].

#### Markers of systemic inflammation

Chronic systemic inflammation, which may partly arise from intestinal microbial translocation, has been proposed to contribute to stunting [27]. Biomarkers of both innate and adaptive immune activation have been used, including cytokines (TNF, IFN-γ, IL-6, IL-10), acute-phase proteins (alpha-1-acid glycoprotein, CRP, ferritin), amino acids (kynurenine, tryptophan) and sCD14, a circulating marker of monocyte activation. Two studies observed associations between linear growth and systemic inflammation [113,124], 2 studies observed conflicting results [70,99], and 2 studies did not observe associations [96,107]. Additionally, two studies found negative associations between systemic inflammatory markers and insulin-like growth factor 1 [99,121]. However, 1 of these 2 studies observed an association in children only at baseline and not at later ages in the study [121]. Four studies included systemic inflammatory markers and linear growth measurements, but did not report associations [53,105,121].

## Discussion

EED has traditionally been defined as a primary gut disorder that initiates a chain of events from intestinal permeability and microbial translocation, via their impact on immune activation, to stunting. In this review, we found that there was fairly little evidence to support the pathway from intestinal permeability to microbial translocation and from microbial translocation to stunting. There was stronger support for the link between intestinal inflammation and systemic inflammation, and between intestinal inflammation and stunting. Other relationships presented conflicting pictures. For example, evidence for or against the role of intestinal damage in intestinal permeability and intestinal permeability/absorption in stunting was evenly split across studies.

These results suggest (i) that the relationships between EED domains are inconsistent; (ii) that the associations between EED domains and stunting are variable, with the best evidence for intestinal inflammation and stunting; (iii) that some domains are harder to measure than others, which may have led to a bias in study findings; (iv) there remains no consistent definition of EED; and (v) the number of studies comparing histopathological analysis of small bowel biopsies to non-invasive biomarkers is lacking. As EED is characterized by small bowel morphological changes, more studies are needed to investigate associations between biopsy samples and a range of non-invasive biomarkers from each domain.

The L:M test has been measured in numerous populations, but procedural details (fasting prior to ingestion, sugar dosage, time of urine collection, assay method) vary, which hinders comparisons between studies; moreover, reference cut-points for the diagnosis of children with EED using the sugar absorption test have not been established [50]. Twelve studies used L:M ratio cut-points of 0.06–0.07 [33,63,101,115,117,125] or 0.10–0.15 [55,108,111,112,103,126] to define EED. Three studies categorized EED as moderate or severe, where L:M values of 0.28–0.45 were used for severe cases [101,104,112]. Seven studies did not report normal L:M cut-point values [32,70,76,96,98,107,114]. The assignment of normal values is challenging as other non-EED related factors can influence the movement of sugars across the epithelium, including age, gastrointestinal motility, variations in gastric emptying, recent diarrhea, mucosal blood flow, and renal clearance [127], resulting in individual and population variability in L:M values. One large multi-site study by Kosek et al compared L:M values to L:M z-values, standardized by age and sex [113]. While L:M values were significantly associated with HAZ up to 15 months, the L:M z-scores were not associated with HAZ at any time points. This study emphasizes the importance of addressing age-specific norms in dual-sugar clearance and illustrates that the assignment of L:M cut-point values may lead to greater risk of EED misclassification in some populations.

Some performance measures, such as the L:M test, may be under- or overestimated in a single population based on its overall characteristics (e.g. higher incidence of diarrhea, prevalence of stunting). For example, two studies of children in rural Malawi found no association between stunting and L:M values [108,111], but each cohort low high mean HAZ values (-2.4 ± 1.3 and -2.8 ± 1.1, respectively) and used high L:M cut-points to classify EED (0.15 and 0.10, respectively). Thus, the values of L:M were likely elevated even in those not classified with EED. Conversely, a third study in Burkina Faso found no association between L:M and stunting, but the cohort was less stunted (mean HAZ = -1.5 ± 1.1) and had a low mean L:M value (0.042; 95% confidence interval: 0.030, 0.072) [101]. The proportionally greater permeability in all subjects with higher L:M values, and the proportionally lower stunting in subjects with lower L:M values, provide evidence in favor of assessing permeability, particularly in cohorts with high variability in the underlying prevalence of stunting.” Moreover, EED may not be a binary (present/absent) condition but a population shift in intestinal structure, wherein some individuals with EED-like characteristics (increased permeability, malabsorption) may not exhibit gut morphological changes. The variability in underlying characteristics presents challenges in interpreting results from heterogeneous study populations.

Finally, lactulose permeability may not accurately reflect the ability of microbes, toxins or larger molecules to pass through the epithelium. The molecular weight of lactulose (342 Da) is significantly smaller than that of antigenic molecules such as LPS (10–20 kDa) [127]. Holes in the tight junction complex that allow paracellular lactulose permeation may not be large enough to allow the movement of microbes or their byproducts across the epithelium. These factors may partly explain why permeability and absorption were not consistently associated with other EED domains, especially inflammation, or stunting.

The association between microbial translocation and stunting was observed in a study of infants in The Gambia [128] over a decade ago, but few studies included in this review support this relationship. Assuming that stunting is a primary outcome of EED, we propose that current tests for microbial translocation may not be appropriate for EED identification. However, strong evidence supports the relationship between intestinal inflammation and stunting, and it is also possible that factors other than microbial translocation may be responsible for the intestinal inflammation observed in individuals with EED. Several investigators [3,113,129] have suggested that pathogen colonization may promote chronic inflammation and induce changes to intestinal microbiota, contributing to EED and stunting, though the exact mechanism is unknown. The intestinal microbiota contains over 500 different species of bacteria, as well as assorted viruses, archaea, fungi and yeasts; the composition and function of the microbiota is largely determined by a combination of dietary, host genetic, inflammatory, clinical (e.g., antimicrobial exposure), and other environmental factors [130]. To our knowledge, only one human study has investigated microbiota dysbiosis in individuals with EED, which was categorised based on L:M ratios [112]. Three of the 6 differentially abundant bacterial genera—*Megasphaera*, *Mitsuokella*, and *Sutterella*—were enriched in children with EED compared to children without EED; the other 3—*Succinivibrio*, *Klebsiella*, and *Clostridium_XI—*were depleted.

Small intestinal bacterial overgrowth (SIBO) may also contribute to intestinal inflammation [131] and EED. A study of urban Bengali children investigated the relationship between the glucose hydrogen breath test, an indicator of SIBO, with EED domains and outcomes [132]. SIBO was not associated with the L:M ratio nor systemic inflammatory markers. However, children with SIBO had significantly worse linear growth and higher concentrations of calprotectin, an intestinal inflammatory marker, compared to those without SIBO. A study of Burmese children observed a similar association between SIBO and linear growth faltering [133] but this association was not present in slum-dwelling children in Brazil [134]. Additional evidence for the relationship between stunting and SIBO is needed.

The inconsistencies highlighted in this review indicate that EED may be more complex than previously conceived. It is possible that EED is not a single entity, but instead a set of phenotypes dependent on unique environmental exposures that vary geographically. The small intestine has a limited repertoire of responses to insult, and enteropathy resulting from multiple potential exposures may have a similar appearance. Some elements of EED may even be adaptive, rather than pathologic, for children living in conditions of poor water, sanitation and hygiene [50]. It is certainly not firmly established that EED is always consequential to linear growth, or indeed that it is definitively associated with stunting. Study subject selection (e.g., including subjects with diarrhea, HIV, or severe acute malnutrition) may influence EED biomarker results. Other potential confounders, including malnutrition, diarrhea, food insecurity, the intestinal microbiota/microbial factors, concomitant treatments (e.g., antimicrobial therapy) that were measured in some studies, but not all, may influence individual study results and our conclusions.

Recent human intervention studies suggest that micronutrient supplementation is not sufficient for EED recovery [115,120,135]; only one study of Zambian adults observed significant improvement in intestinal morphology after micronutrient supplementation [77]. Morphological studies are more feasible in animals than humans and have the potential to reveal key associations that are otherwise difficult to assess in human studies. An experimental study to evaluate the effects of malnutrition and exposure to Bacteroidales and *E*. *coli* in mice observed that malnourished mice with bacterial exposure experienced increased permeability and tail length (a surrogate for length of mouse), as well as blunted intestinal villi characteristic of EED [136]. Mice with a malnourished diet—regardless of bacterial exposure—also had greater microbiota dysbiosis and intestinal permeability, but without blunted intestinal villi. Mice with bacterial exposure but normal diets experienced normal tail growth and no change in permeability. These results suggest an interaction may exist between malnutrition and the presence of specific bacterial species, where EED features are present only in individuals with both. It should be noted that this study used mouse tail length as a surrogate for mouse length; how these findings relates to human linear growth remains unclear.

This review summarizes evidence for each pathway in a consensus framework model of EED (and its hypothesized component/contributing domains) and stunting. Information from a wide range of published articles representing very different biomarker measurement methods, heterogeneous study populations, and different analysis and reporting methods, was reviewed. We did not assess study quality in this review given the heterogeneity, nor did this review include studies of EED *in vitro* and *in vivo* models. Many studies did not report associations between domains; these missing or non-reported data may have influenced our conclusions. This is a consistent feature of EED-related studies, as already highlighted by Denno et al in their review of biomarkers from 2000–2010 [29,50]. The studies included in that review, which categorized biomarkers into eight EED domains, provided conflicting evidence for the links between permeability and linear growth [119,137–142], permeability and intestinal inflammation [137,143], as well as systemic inflammation and linear growth [138,140]. Microbial translocation was not included as a domain in their review, but one study reported a positive association between IgG endotoxin-core antibody and permeability [137]. No conclusive set of biomarkers for EED diagnosis were identified in either the 2000–2010 or in our review of the literature from 2010–2017.

The domains and specific pathways defined and investigated in our review are open to debate; however, we focused on pathogenic processes that have been long been hypothesized to be part of EED. Other potential domains (e.g. digestion, microbial drivers) have been identified within EED pathways [3,35–37], but these domains were minimally reported or missing from selected studies and were not included in our results.

### Conclusions

In this review, we evaluated individual pathways between domains within EED and between each domain and stunting using studies published between 2010–2017. We found evidence to support the link between intestinal and systemic inflammation and stunting, but little support for the link between microbial translocation and stunting within the limits of current tests. There was conflicting evidence for the associations between intestinal damage and intestinal permeability, as well as intestinal damage and stunting. These results suggest that current biomarkers and proposed mechanisms of EED pathogenesis may need reconsideration, and future studies of pathogen-induced changes to the intestinal microbiota should investigate alternative pathways of the effect of intestinal and systemic inflammation on growth in children.

## Supporting information

S1 Table Standardized data abstraction form. (DOC)

S2 Table PRISMA checklist. (DOC)
