# Supplementary material for: Environmental enteric dysfunction pathways and child stunting: A systematic review
Source: PLoS Negl Trop Dis. 2018 Jan 19;12(1):e0006205. doi: 10.1371/journal.pntd.0006205 (PMC5792022; doi:10.1371/journal.pntd.0006205)
Supplement: S1 Table — (DOC) [file pntd.0006205.s001.doc]

| **Reference and Study Outcomes of Interest** | **Location and Target Population** | **Design and Sample Size** | **Measurements** | **Results** | **Discussion/Conclusions** | **Comments** |
| --- | --- | --- | --- | --- | --- | --- |
| 2017  **Brown G et al**  Linking growth and gut function: Environmental enteropathy in children under 2 years in rural Nicaragua  To explore the relationship between intestinal function and growth outcomes in children under 2 years | Rural Nicaragua  Children (mean age = 12.04 mo) | Cross-sectional  n=53 | Anthropometric  HAZ, WAZ, BAZ  Permeability  L:C | Anthropometric  11% were stunted (HAZ < −2)  2% were wasted (BAZ < −2).  Permeability  Median L:C ratio = 0.10 (IQR = 0.16)  L:C ratio was not significantly correlated with WAZ or BAZ  Negative correlation between HAZ and L:C ratio | Children with higher HAZ (HAZ > 0) had worse gut function (p = 0.0492) | *Abstract only*  No definition of EED provided |
| 2017  **Campbell RK et al**  Biomarkers of environmental enteric dysfunction among children in rural Bangladesh  To evaluate convergence among biomarkers and describe risk factors for EED. | Rural Bangaldesh  18 mo children | Cross-sectional nested in a community-based RCT of complementary food supplements  n = 566 eligible  n = 539 final  (27 children refused EED assessment) | Anthropometric  LAZ, WAZ  Permeability  2-h sugar absorption test, dosed by body weight (2-20mL/kg with 255 mg/mL L and 50 mg/mL M)  Intestinal damage and repair  GLP-2  Intestinal inflammation  MPO, AAT, NEO combined to make gut inflammation (GI) score  Systemic inflammation  AGP  CRP  Total IgG, IgM, IgA  Microbial translocation  EndoCAb IgG, IgM, IgA | Anthropometric  - LAZ and WLZ at 6 mo and their change from 6 to 18 mo were associated only with the gut permeability score (L:M ratio)  - Lower GLP-2 was associated with higher ∆LAZ  - Higher EndoCAb positively associated with ∆LAZ  Permeability  - Mean L:M: 0.06 [0.05, 0.06]  - Elevated in 39% of children  - Correlation of L:M with other biomarkers were not significant  - A multivariate model including all biomarkers only explained 10.8% of the observed variability in L:M  - MPO was modestly correlated with AAT (r=0.33, p<0.01)  Intestinal inflammation  - Concentrations were elevated in 84% (MPO), 56% (AAT), and 100% (NEO) of children  Systemic inflammation  - Concentrations were elevated in 56% (AGP) and 20% (CRP) of children  - CRP was correlated with AGP (r=0.55, p<0.01)  Microbial translocation  - EndoCAb values positively correlated with LAZ values  - EndoCAb and Igs were consistently intercorrelated  - EndoCAb inversely associated with GLP-2 | Serum and stool biomarkers demonstrated low agreement internally and with L:M test, and principal component analysis (PCA) and partial least squares (PLS) regression did not identify a subset of markers that closely approximated L:M.  EndoCAb – LAZ results were opposite of what is expected | Definition: subclinical condition of partial villous atrophy, crypt hyperplasia, leaky tight junctions and enteric immune cell proliferation  Cutoffs  *L:M > 0.07*  *MPO > 2000 ng/mL*  *AAT > 270 μg/mL*  *NEO > 70 nmol/L*  *AGP > 1 g/L*  *CRP > 5 mg/L* |
| 2017  **Kosek M et al**  Causal Pathways from Enteropathogens to Environmental Enteropathy: Findings from the MAL-ED Birth Cohort Study  To assess the role of enteropathogens and other factors in growth faltering from birth to two years | Eight sites:  Bangladesh (BGD)  India (INV)  Nepal (NEB)  Pakistan (PKN)  Brazil (BRF)  Peru (PEL)  South Africa (SAV)  Tanzania (TZH)  Children 0-2 yo, enrolled within 17 days of birth  Exclusion:  - birth weight < 1500 g  - were very ill  - non-singleton  - mother was < 16 yo | Longitudinal cohort  n = 1253  (complete samples = 744) | Anthropometric  LAZ, WAZ  Permeability  L:M ratio  LMZ score  AAT  Intestinal Inflammation  NEO, MPO  Systemic Inflammation  AGP | Anthropometric  - No statistical support for LMZ relating to changes in either LAZ or WAZ  - At age 1, negative association between MPO and ∆LAZ and ∆WAZ at age 1  - At both ages, negative association between AAT and ∆WAZ  - *Giardia* was associated directly with reduced growth, but not with the fecal biomarkers.  - Higher AGP concentrations were associated with decreased ∆LAZ at both ages and ∆WAZ at age 1.  Permeability  - LMZ tended to be higher in children with pathogens detected, especially *Cryptosporidium* and *Giardia*  - At age 1, AAT was positively associated with LMZ; MPO was negatively correlated with LMZ  Intestinal Inflammation  - In a model containing all pathogen groups, all biomarkers, and LAZ and WAZ, Group II bacteria showed positive association with MPO at both ages  - Group III bacteria were associated with lower AAT at age 1 and higher MPO at age 2  Systemic Inflammation  - At age 2, MPO and AGP were positively correlated; NEO was negatively correlated with AGP | Enteropathogens changed the concentration of NEO, MPO, AAT, and LMZ  Enteropathogens and gut inflammation both relate to systemic inflammation  Enteropathogens present even in the absence of diarrhoea  According to these biomarkers, EED contributes to stunting but is not the biggest contributor  No association between intestinal permeability (as measured by L:M) and future growth  -- AAT may be a more valid measure of EED  Reductions in exposure to pathogens could reduce systemic inflammation (as measured by AGP) and subsequent stunting | Definition: Long-term effects on child health, stemming from host response to frequent enteric infections that alter the gut, even in the absence of diarrhoea or acute gastrointestinal illness |
| 2017  **Lee G et al**  Environmental enteropathy is associated with cardiometabolic risk factors in Peruvian children  To assess the association of EED with the cardiometabolic profile among young children living in an environment of intense enteropathogen exposure | Peru site within the MAL-ED study  Children 3-5 yo  Eligible: children living a minimum of 18 mo and were still living in the study area | Case-cohort  n = 303 enrolled  n = 164 eligible  n = 156 final  n = 154 EndoCAb  n = 155 apolipoprotein profile | Permeability  2-h sugar absorption test  20 ml dose of L:M solution, food provided after 30 minutes  %L, %M, L:M ratio  Anthropometric  HAZ, WAZ, WHZ  Cardiometabolic factors  Glucose  TG  TC  HDL-c  LDL-c  VLDL-c  HOMA-IR  Intestinal Inflammation  MPO  NEO  AAT  Microbial translocation  EndoCAb  Systemic Inflammation  AGP  CRP | Permeability  %L and %M strongly positively correlated  %M positively associatedwith HDL-c, Apo-AI and Apo-B  %M negatively associated with VLDL-c, the TC/aHDL-c ratio, the Apo-B/Apo-AI ratio, HOMA-IR, high TG and high blood pressure  No consistent associations between L:M ratio measured at any time and the cardiometabolic profile  By 15 months of age:  - 46% had L:M ratios above the 90th percentile for a healthy tropical reference population  - 70% had subclinical inflammation (defined by AGP)  Anthropometric  No associations between biomarkers and stunting were reported  Microbial translocation  No association between EndoCAb and cardiometabolic profile  No correlation reported between EndoCAb and L:M  Systemic Inflammation  At 7 months, associations between:  High blood pressure—CRP (OR=1.58)  High blood pressure—AGP (OR=1.57) | Study supports the potential for a relationship between EED and the cardiometabolic profile through both inflammatory and non-inflammatory mechanisms  Limitations include relatively small sample size and that metabolic syndrome cannot be assessed at such an early age | Definition: syndrome of altered small intestine structure and function characterized by partial villus atrophy and crypt hyperplasia  Cutoffs:  *L:M > 0.07*  *%L > 0.08%*  *%M > 0.86%*  *EndoCAb < 75GMu/ml*  *AGP>1 g/l* |
| 2017  **Ordiz et al**  Environmental enteric dysfunction and the fecal microbiota in Malawian children  Use 16S rRNA gene sequencing to identify phylogenetic groupings of bacteria to detect dysbiosis associated with EED | Rural Malawi  12-23 mo healthy children  Exclusion: diarrhoea in previous 7 days | Cross-sectional nested in RCT  n = 81 | Anthropometric  HAZ  Permeability  L:M  Bacterial genera | Children with EED at baseline: n (%)   - No EED: 8 (10) - Moderate: 49 (60) - Severe: 24 (30)   Anthropometric  Baseline categorization of EED (i.e. baseline L:M ratio) was associated with ∆HAZ in the subsequent 3 months (P = 0.01, Tukey-Kramer multiple comparison test)  Bacterial genera presence   - 2 genera present only in noEED individuals - 51 in moderate EED - 28 in severe EED; - 138 in all individuals regardless of EED status - 6 genera were differentially abundant in EED vs. no EED: - *Megasphaera*, *Mitsuokella*, and *Sutterella* were more prevalent in EED - *Succinivibrio*, *Klebsiella*, and *Clostridium_XI* were less prevalent in EED | EED is not profoundly associated with fecal dysbiosis,but six genera were identified as having significantly different abundances in EED.  Study is limited by the use of fecal (colonic) bacterial colonies opposed to small bowel colonies and therefore cannot determine a causal relationship between EED and bacterial genera present | Definition: characterized bysmall bowel villous atrophy and inflammation  EED Cutoff  No EED (< 0.15)  Moderate (0.15 – 0.45)  Severe (L:M > 0.45) |
| 2017  **Semba R et al**  Environmental Enteric Dysfunction is Associated with Carnitine Deficiency and Altered Fatty Acid Oxidation  Use an LC-MS/MS metabolomics platform to comprehensively assess metabolites related to carnitine deficiency in individuals with EED | Rural Malawi  Children 12-59 mo  Exclusion: presence of congenital or chronic disease or caretaker-reported diarrhoea, or were under treatment for acute malnutrition | Cross-sectional  n = 400  n = 323 (80.7%) with EED as measured by L:M ratio | Anthropometric  HAZ, WAZ  Permeability  4-h dual sugar absorption test, 1g M: 5g L  L:M ratio  Serum metabolites | Anthropometric  HAZ, mean ± SD (p = 0.25):   - EED: -2.3 ± 1.3 - no EED: -2.5 ± 1.3   WAZ, mean ± SD (p = 0.29):   - EED: 0.3 ± 1.0 - no EED: 0.1 ± 1.3   Stunted (p = 0.31):   - EED: n= 52 (68%) - no EED: n = 198 (61%)   Serum metabolites  77 metabolites were significantly correlated with L:M ratio to classify EED status:   - 21 negatively associated - 56 positively associated   These 77 metabolites had only a modest ability to discriminate between children with and without EED, as determined by multiple machine-learning classification algorithms  Children with increased gut permeability had *elevated* serum levels of:   - concentrations of four odd-chain fatty acids - 3-hydroxy-3-methylutarate - 3-aminoisobutyrate - trimethylamine-*N*-oxide - S-adenosylhomocysteine - cystathionine - taurine - phenylacetylglutamine - 4-hydroxyphenylacetyl-glutamine - phenylacetate - serotonin - homocitrulline - and more   Children with increased gut permeability had *reduced* serum levels of:   - hippurate - 3-hydroxyhippurate - dietary polyphenolic compounds (intermediates in the pathways to hippurate) - dihydroferulic acid - catechol sulphate - citrulline - ornithine - three fat-soluble substances - and more | This study shows that EED (as measured by L:M ratio) is associated with secondary carnitine deficiency.  Gut permeability (L:M ratio) was associated with a serum metabolite profile corresponding to elevated serum concentrations of:   - 9 acylcarnitines - 3 intermediate metabolites associated with blocked β-oxidation of fatty acids - 4 metabolites related to upregulation of ω-oxidation of fatty acids   Children with EED may havealtered metabolism associated with an abnormal gut microbiome, as demonstrated by reduced levels of hippurate, which is normally strongly associated with diet and gut microbiome  Limitations: Cross-sectional study cannot show that the relationship between ED and carnitine deficiency is causal | Definition: asymptomatic condition characterized by chronic inflammation of the duodenum and jejunum and abnormal gut permeability  Cutoff  *EED: L:M ≥ 0.15* |
| 2017  **Semba R et al**  Environmental enteric dysfunction is associated with altered bile acid metabolism  To examine the potential relationship between EED and abnormalities in serum bile acid metabolism. | Rural southern Malawi  Children 12-59 mo  Exclusion: presence of congenital or chronic disease or caretaker-reported diarrhoea, or were under treatment for acute malnutrition | Cross-sectional  n = 313 | Permeability  4-h dual sugar absorption test, 1g M: 5g L  L:M ratio  Anthropometric  HAZ, WAZ  Serum bile acids | n = 244 (80%) with EED as measured by L:M ratio  Anthropometric  HAZ, mean ± SD:   - EED: -2.4 ± 1.3 - no EED: -2.4 ± 1.3   WAZ, mean ± SD:   - EED: 0.2 ± 0.9 - no EED: 0.3 ± 0.9   Stunted (p = 0.31):   - EED: n= 52 (68%) - no EED: n = 198 (61%)   Serum bile acids  Total serum bile acids were significantly lower in children with EED vs. no EED(P = 0.0009)  3 most abundant serum bile acids were GCDCA, GCA, and GDCA; none of these were significantly different between EED vs. no EED groups  Serum TCDCA, TMCA, and GUDCA were less abundant but were significantly different in EED vs. no EED groups  Serum bile acid ratios, which provide the relative proportion of bile acids conjugated by taurine, were higher in children with EED vs. no EED (P < 0.0001) | The present study suggests that children with EEDhave alterations in bile acid metabolism. Total serum bile acids were approximately 12% lower in children with EED vs. no EED.  Malawian children had a lower proportion of TCDCA compared with studies of healthy Austrian children  Serum concentrations of unconjugated bile acids were not differentbetween children with and without EED, which suggests that smallbowel bacterial overgrowth is not a significant problem in this population | Definition: clinically asymptomatic condition characterized by inflammation of the smallbowel mucosa, villous atrophy, malabsorption, and increased intestinal permeability  Cutoff  *EED: L:M ≥ 0.15* |
| 2017  **Wang AZ et al**  A Combined Intervention of Zinc, Multiple Micronutrients, and Albendazole Does Not Ameliorate Environmental Enteric Dysfunction or Stunting in Rural Malawian Children in a Double-Blind Randomized Controlled Trial  To assess improvements in EED (as measured by L:M and linear growth) after 12-24 weeks of combined intervention of zinc, abendazole, and multiple micronutrient powder | Rural Malawi  12-35 mo children who were not acutely ill, including twins  Exclusion: inability to provide a complete 4-h urine collection, recent history of >3 loose stools/day, SAM, moderate acute malnutrition, or chronic illness | RCT  n = 263 screened  n = 254 randomly assigned to intervention groups  n = 241 successfully completed all 3 dual-sugar absorption tests | Permeability  4-h L:M sugar absorption test  5g L: 1g M  Anthropometric  Length, weight | Intervention group  Initial L:M ratio: 0.33  ∆ L:M ratio after 24 wk: 0.088  Initial length (cm): 80.9  Length 24 wk: 85.3  Placebo group  Initial L:M ratio: 0.34  ∆ L:M ratio after 24 wk: 0.080  Initial length (cm): 80.4  Length 24 wk: 84.9 | No significant differences between placebo and intervention groups with regards to L:M ratio or linear growth | Definition: subclinical condition characterized by diffuse blunting of intestinal villi and inflammation of the lamina propria  ∆ L:M ratio ≥ 0.06 is the smallest interval change that would be statistically significant |
| 2016  **Arndt M et al**  Fecal markers of environmental enteropathy and subsequent growth in Bangladeshi children | Bangladesh site of MAL-ED study  Children 0-24 mo  Exclusion:  - maternal age of < 16 years  - not a singleton pregnancy  - another child already enrolled in the MAL-ED study  - severe disease requiring hospitalization before recruitment  - severe acute or chronic conditions diagnosed by a physician | Longitudinal  n = 246  (5.7% LTFU before 12 mo, 14.6% LTFU before 24 mo) | Anthropometric  WAZ, LAZ, WHZ  Intestinal Inflammation  AAT, MPO, NEO: combined to form Kosek EE disease activity score | Anthropometric  At birth, ~16% of children were stunted (LAZ < –1)   - After 24 months, 78% of those stunted at birth were still stunted - After 24 months, 44% of those not stunted at birth were stunted   Intestinal Inflammation  Samples considered normal in non-tropical settings:   - 63% AAT - 68% MPO - 95% NEO   The Kosek EE score was negatively associated withLAZ in months 1-3 and 12–21, but not in months 3–9.  A one-unit increase in EE score in months 3-21 was associated with a decrease of 0.009 LAZ per 3-month period (95% CI: -0.018, 0.000)  A one-unit increase in EE score in months 12-21 was associated with a decrease of 0.013 LAZ per 3-month period (95% CI: -0.023, -0.004)  Note: When the significance level was adjusted for multiple comparisons (using either the Benjamini–Hochberg or Bonferroni method), none of the marker coefficients remained significant. | High fecal MPO levels were associated with decreases in 3-month linear growth in the second year of life.  Neither AAT nor NEO were associated with subsequent growth.   - The composite EE score appears to be driven by MPO levels   None of the fecal markers tested nor the EE score were significantly associated with subsequent 6-month growth | Definition: subclinical intestinal disorder characterized by mucosal and systemic inflammation, reduced intestinal barrier integrity, bacterial translocation, and reduced intestinal absorptive capacity  Normal cutoff for non-tropical settings  *AAT < 0.27 mg/g*  *MPO < 2,000 ng/ mL*  *NEO < 70 nmol/L*  Kosek score ranges from 0-10 |
| 2016  **Donowitz et al**  Small intestine bacterial overgrowth and environmental enteropathy in Bangladeshi children  (1) To determine the prevalence of SIBO in Bangladeshi children and its association with malnutrition; (2) To determine the association of SIBO with sanitation, diarrheal disease, and EED  (Part of PROVIDE study) | Urban Bangladesh  Children 2 yo  Exclusion:  WAZ < 3 SD | Cross-sectional  n = 103 assessed for SIBO testing  n = 90 successfully completed SIBO testing | Anthropometric  LAZ  SIBO  Glucose hydrogen breath test  Permeability and Absorption  L:M ratio  Intestinal damage and repair  REG1B  Intestinal inflammation  calprotectin  Systemic inflammation  CRP, 17-plex Luminex cytokine panel, INFg, IL-1B, IL-2, IL-4, IL-5, IL-6, IL-7, IL-8, IL-10, IL-12, IL-13, IL-17, MCP-1, MIP-1B, TNFa | SIBO  - Children with SIBO had significantly worse linear growth (stunting) from enrolment to age 2 compared to those without SIBO  - Diarrheal disease, WAZ, SES were not significant predictors of SIBO  Intestinal damage and repair  REG1B was associated with SIBO  Intestinal inflammation  Calprotectin was significantly increased in children with SIBO  Permeability and Absorption  No significant different in the L:M ratio for children with or without SIBO  Systemic inflammation  None of the markers were significantly different between the two groups | Findings are contradictory to other investigations which found an increase in intestinal permeability in adults with SIBO in the developed world  Lack of L:M ratio differences may indicate that SIBO and EE are separate but concomitant conditions or that EE is actually a syndrome encompassing a heterogeneous group of environmentally-derived intestinal inflammatory conditions with variable manifestations but common outcomes | Definition – an inflammatory intestinal disorder of the developing world that has been implicated in growth failure and poor neurocognitive out- comes  Cutoff  Normal SIBO < 12-ppm over baseline |
| 2016  **Faubion WA et al**  Improving the detection of EED: a lactulose, rhamnose assay of intestinal permeability in children aged under 5 years exposed to poor sanitation and hygiene  To address the limitations of the current L:M test and to assess the intestinal barrier function in infants with EED using the LR test | USA  Children 2-13 mo  Zambia  Children 2-36 mo  Peru  Children 15-29 mo  Exclusion:  concurrent illness, had diarrhea within 1 month, had been given antibiotics within 1 month | Case-control  n = 131 total  USA (n=27)  Zambia (n=85)  Peru (n=19) | Anthropometric  HAZ, WAZ  Permeability  1-h L:R test  - 1000mg L: 22mg R  %L, %R | Anthropometric  Median (range) WAZ and HAZ:   - Peru: (WAZ: −0.8 (−2.7, 0.8); HAZ: −1.8 (−3.3, −0.2)) - Zambia: (WAZ: −1.4 (−4.0, 1.1); HAZ: −2.3 (−8.5, 1.2)) - USA: (WAZ: 0.5 (−0.4, 2.1); HAZ: −0.1 (−1.8, 2.4))   HAZ and WAZ in Peruvian and Zambian cohorts were significantly different than the US cohort (p < 0.001), indicating presence of EED in the two populations  WAZ and HAZ as outcome variables:   - %L significantly predicted both WAZ (p < 0.001) and HAZ (p = 0.0018) - %R did not significantly predict either WAZ or HAZ - L:R significantly predicted WAZ (p = 0.013) but failed to predict HAZ (p = 0.098)   Permeability  Feasibility of LR test is indicated by completion of tests:   - Peru: 19/19 (100%) - Zambia: 73/85 (86%) - USA: 22/27 (81%)   Detectable concentrations of mannitol were present in over 90% of children in all three cohorts within the baseline urine sample, while presence of rhamnose was negligible (2.6%).  LR ratio:   - Peru: (0.75 (0.15, 5.02)) - Zambian (2.26 (0.08, 14.48)) - USA: (0.14 (0.06, 1.00))   Significant differences between Peruvian and Zambian cohorts compared to US cohort (p<0.001)  Percent (n/N) of children displaying abnormal LR ratio (compared to 95^th^ centile from the US population as the upper limit of normal):   - Peru: 53% (1/22) - Zambian 82% (10/19) - USA: 5% (60/73) | Authors speculate that the reduced %R may be due to decreased intestinal SA, therefore recovery of lactulose alone may potentially be a more accurate marker of intestinal permeability than %R alone, LR ratio or L:M ratio  Low R^2^ values for both WAZ and HAZ regression models indicated that there are likely other factors that contribute to impaired growth | Definition: disorder of the small bowel that is characterized by chronic intestinal inflammation, villous atrophy, malabsorption and linear growth failure |
| 2016  **Guerrant R et al**  Biomarkers of environmental enteropathy, inflammation, stunting, and impaired growth in children in northeast Brazil  1. To examine potential fecal, urinary and plasma biomarkers to determine how they associate with each other and with malnutrition  2. To determine which biomarkers best predict impaired subsequent growth | Northeastern Brazil  Children 6-26 mo  Exclusion:  - underlying disease  - parent/guardian < 16 yo | Case-cohort, matched on gender and age  n = 402 recruited  n = 375 enrolled, provided initial measurements  n = 301 final | Anthropometric  HAZ, WAZ  Permeability/absorption  L:M, %L, %M  AAT  Intestinal damage and repair  REG1  i-FABP  Bacterial translocation  plasma LPS  IgG anti-LPS/anti-FliC  IgA anti-LPS/anti-FliC  Tight junction impairment  zonulin  Cdn-15  Intestinal Inflammation  MPO  NEO  Systemic inflammation  plasma SAA  sCD14  LPC  citrulline  tryptophan  kynurenine | Anthropometric  Significant associations between:   - Lower WAZ with IgA anti-LPS, i-FABP at baseline - Lower HAZ with IgA anti-FLA and anti-LPS, i-FABP, citrulline, SAA at baseline - HAZ/WAZ with zonulin, children > 12 mo - growth impairment correlated with higher LPS, I-FABP and SAA - citrulline levels and weight gain primarily in girls (p < 0.001) - tryptophan and growth in boys (p = 0.010) - For urine L:M, higher values correlated (controlling for age and gender) with impaired growth (∆HAZ) (r = -0.173; p = 0.009; n = 230). - Subsequent growth was worse in those with higher AAT   Biomarkers  Significant associations:   - Cdn-15 with negative L:M, AAT, REG1, lipocalin-2 - KTR with MPO (p < 0.01) - KTR with CRP (p < 0.01) - KTR with citrulline, negative (p < 0.01) - MPO with AAT, LPC, REG1, kynurenine, KTR (p < 0.01) - MPO with L:M, %L, and tryptophan (negative) (p < 0.05) - REG1 correlated with AAT, MPO, and NEO (p < 0.01) - REG1 with LPC and lactoferrin (p < 0.05) - MPO with CRP, kynurenine, and KTR (p < 0.01), - MPO with SAA and tryptophan (negative) (p < 0.05) - interaction between MPO and NEO   Principal Component Analysis (PCA), identifying groups of biomarkers that relate to subsequent growth:   - B1: LPS (translocation) - B2: L:M and i-FABP (disrupted absorptive function and epithelial cell damage) - B3: further barrier disruption with tight junction modulation (e.g. REG1 and AAT with zonulin)   Significant associations:   - PCA B2 with MPO - PCA B1 with baseline HAZ - B3 with MPO - B3 with NEO   Significant predictors of growth impairment (∆HAZ), controlling for age and gender:   - HAZ at baseline (p < 0.001) - MPO (p = 0.041) - PCA B2 | Many biomarkers are present in already stunted children, namely systematic IgA with anti-LPS and anti-FLA and i-FABP  Other biomarkers may help predict future stunting, including MPO, AAT, L:M, LPS, i-FABP, and SAA, as well as the interaction between MPO and NEO  Boys and girls had slightly different biomarkers, which may be due to the higher prevalence of stunting in males of the lowest SES  Biomarkers tend to cluster into 3 main groups: (1) intestinal translocation, (2) intestinal mucosal barrier disruption and inflammation, and (3) systemic inflammation  Barrier function, intestinalinflammation and systemic markers are linearly associated with each other as well as baseline stunting and ∆HAZ  Cdn-15 has never been used to measure healthy gut function in individuals with EED; Cdn is inversely correlated with L:M, AAT, lipocalin-2, and REG1 and may be used as a positive biomarker of gut health  PCA analysis revealed that biomarkers of barrier disruption have significant correlation with baseline HAZ and ∆HAZ, as well as with multiple other groups of biomarkers  Conclusion: fecal MPO and AAT, and L/M, plasma LPS-neutralizing activity,I-FABP, SAA, CRP, citrulline and tryptophan provide the most promise for measuring subsequent growth failure | Definition: damage of villous architecture, caused by disruption of gut barrier function, passage of microorganisms and/or their bioproducts from the intestinal lumen to the lamina propria, and mucosal inflammation |
| 2016  **Kelly P et al**  Endomicroscopic and Transcriptomic Analysis of Impaired Barrier Function and Malabsorption in Environmental Enteropathy  To assess the association between CLE biopsy data | Southern Zambia  Adult volunteers recruited by door-to-door invitations, focus group discussions, and individual interviews  Excluded: individuals with concurrent illness, pregnancy, use of NSAIDS or antibiotics within one month, or recent helminth infection | Cross-sectional  n=49 total  n=35 HIV seronegative  n=14 HIV seropositive   - n=6 on ART - n=8 not on ART   *ART = antri-retroviral therapy | Anthropometric  height, weight, mid upper arm circumference  Morphometry  biopsy samples  Permeability:  Confocal laser endomicroscopy (CLE) for biopsy  Microbial translocation:  LPS  LPS binding protein  Intestinal damage and repair  i-FABP  GLP-2  Mineral absorption:  Zinc uptake  Systemic Inflammation  CRP  sCD14  CD163  Intestinal Inflammation  AAT  Transcriptomics | Permeability  Watson scores:   - 1: n=4 (10%) - 2: n=6 (15%) - 3: n=31 (76%)   Plumes were positively associated with i-FABP  Morphometry  All biopsies contained villous blunting, crypt lengthening, and lamina propria inflammation  No significant differences between HIV and non-HIV individuals  Microbial translocation:  LPS was detectable in all samples  LPS was associated with:   - GLP-2 (β = -0.13, n = 43; P = 0.007) - cell shedding (β = 0.83, n = 43; P = 0.035) - epithelial perimeter (β = 10136; P = 0.01) - villus SA:volume ratio, fractional polynomial model (β = 59006; P = 0.007) - villus SA:volume ratio, linear regression model (ρ = 0.46; P = 0.003)   LPS was not associated with CRP, sCD14, CD163, or LBP  Mineral absorption  21/48 (44%) participants were deficient in zinc  Zinc was positively associated with GLP-2 (β = 2.70; P = 0.03)  Transcriptome analysis (n=8)  n=4, very low/undetectable plumes  n=4, frequent plumes  23 differentially expressed genes between the two groups   - Down-regulated genes included those involved in metal ion uptake, anti-protease activity, mucosal protection, and host defense - Up-regulated genes included those involved with inflammation | The U-shaped relationship between translocation and villus SA suggests that the reduced villus surface area in enteropathic disorders may act at least in part to mitigate the translocation burden in the presence of epithelial defects  No correlation between zinc uptake and morphometric attributes, but severe enteropathy was associated with reduced expressed of DMT-1, which regulates zinc uptake  Low circulating GLP-2 concentrations were associated with very high levels of translocation; may indicate primary endocrine failure to synthesize GLP-2 | Definition: an asymptomatic disorder characterized by variance in the small intestinal mucosal architecture  Watson Score  *1 = no fluorescein plumes / leakage*  *2 = fluorescein leakage but no microerosions*  *3 = fluorescein leakage with any microerosions*  *Zinc deficiency = plasma concentrations below 11 μmol/l* |
| 2016  **Kosek M et al**  Plasma tryptophan and the kynurenine-tryptophan ratio are associated with the acquisition of statural growth deficits and oral vaccine under-performance in populations with environmental enteropathy  To determine whether KTR, citrulline, and cytokines were associated with growth deficits in infancy and early childhood | Rural Peru, rural Tanzania arms within the MAL-ED study  Children 0-2 yo, enrolled within 17 days of birth  Exclusion:  - birth weight < 1500 g  - were very ill  - non-singleton  - mother was < 16 yo | Longitudinal Cohort  n = 494 (including those LTFU over 24 mo)  Peru  n = 303 enrolled  n = 262 with one measurement of K, T, citrulline  n = 198 retained for 24 mo  Tanzania  n = 262 enrolled  n = 219 with one measurement of K, T, citrulline  n = 211 retained for 24 mo | Anthropometric  LAZ, WAZ, WHZ  Mucosal Mass  Citrulline  Permeability  L:M ratio  Systemic Inflammation  KTR*  Plasma cytokines:   - AGP - CRP (Peru only) - IFN- γ (Peru only) - IL-10 (Peru only) - IL-6 (Peru only) | Anthropometric  Peru:   - Mean LAZ decreased from -1.3 to -1.9 over 24 mo - At 24 mo, ~40% of children - 1-SD increase in tryptophan was associated with a gain in LAZ of 0.10 over the next 6 months (95% CI: 0.05–0.16, P < 0.001) - At 3 months, 1-SD increase in citrulline concentration was associated with a gain in LAZ of 0.19 over the next 6 months (95% CI: 0.07–0.31, P =0.002);   Tanzania:   - Mean LAZ decreased from -1.0 to -2.7 over 24 mo - At 24 mo, ~71% of children - 1-SD increase in tryptophan was associated with a gain in LAZ of 0.13 over the next 6 months in Tanzania (95% CI = 0.03–0.22, P = 0.009) - Citrulline was not associated with growth   Neither kynurenine nor the KTR demonstrated significant relationships with LAZ    Mucosal Mass  Mean citrulline level: 21.1 μM (95% CI: 12.7–32.8)  Citrulline and LAZ were negatively associated at 3 mo (ρ = −0.16, P = 0.01) but not associated at any other time  Citrulline was not associated with LAZ, WAZ, or WHZ at any other time.  Permeability  %L was not associated with kynurenine or tryptophan, but was negatively associated with citrulline levels  %M was associated with higher tryptophan and kynurenine, but not associated with citrulline  Systemic Inflammation  Mean concentrations:   - Kynurenine: 2.9 μM (95% CI: 1.8–4.5) - Tryptophan: 49.9 μM (95% CI: 22.1–74.9) - KTR: 60.6 μM (95% CI = 38.1–121.8) - AGP: 113 (95% CI: 73-177)   In Peru, tryptophan and KTR were not associated with LAZ, WAZ, or WHZ at any time.  Kynurenine and LAZ were negatively associated at 3 mo (ρ = −0.16, P = 0.03) but not associated at any other time  In Tanzania, LAZ and WAZ were moderately inversely associated with KTR at 7, 15, and 24 mo   - Spearman’s ρ for LAZ = −0.3, P < 0.0001 - Spearman’s ρ for WAZ = −0.25, P < 0.0001   Mean levels of cytokines (Peru only):   - CRP: 1.05 mg/L (95% CI: 0.14–12.0) - IFN- γ: 3.08 pg/mL (95% CI: 1.45–12.18) - IL-10: 5.53 (95% CI: 3.25–10.82) - IL-6: 1.28 (95% CI: 0.62–4.61)   Correlation of CRP with AGP (95% CI = 0.54–0.68, P < 0.01) and CRP with IL-6 of similar magnitude  Strong correlation of KTR with IFN-γ (0.24–0.39, P < 0.01) and KTR with IL-10 (0.27–0.38, P < 0.01) | Tryptophan concentrations are associated with subsequent improved linear growth in children in Peru and Tanzania when measured at 3, 7, 15, and 24 mo  The relationship between tryptophan, KTR, and linear growth is stronger than that of CRP, AGP, and IL-6. This may suggest that tryptophan may be a more specific and predictive prognostic marker than these more common markers of systemic inflammation.  Tryptophan concentrations are influenced by dietary intake; given the relationship between tryptophan and growth, it may be possible to improve growth by diet diversification or by introducing enriched foods, such as protein maize, at a young age  Alternatively, intestinal microbiota—which are known to produce tryptophan and its metabolites—may play important roles in maintaining optimal tryptophan concentrations  KTR was largely driven by high tryptophan levels, but was exacerbated by low kynurenine levels  Citrulline was not a good predictor of linear growth deficits, but were inversely associated with measures of systemic inflammation (CRP, AGP, and IL-6) at all ages; this provides evidence that enterocyte function, intestinal infection, and systemic immune activation are closely linked in EED | Definition: functional disorder of thegut resulting from multiple enteric infections which result in chronic intestinal immune activation, augmented intestinal permeability, and persistent systemic immune activation |
| 2016  **McDonald CM et al**  Elevations in serum anti-flagellin and anti-LPS Igs are related to growth faltering in young Tanzanian children  To assess whether LPS- and FLA-specific immunoglobulins concentrations were associated with poor growth in children at risk of EED | Tanzania  Infants 0-18 mo  Exclusion of exposed: congenital anomalies or other conditions that would interfere with study procedures  Exclusion of unexposed:  LAZ < -2 or > 2; WAZ < -2 or > 2; no growth data were available for the review and analysis; evidence of a recent fever, (>38.58°C in the past 48 h); evidence of a systemic illness | Longitudinal cohort within RCT  n = 590 exposed, Tanzania  n = 36 unexposed, Boston Children’s Hospital  (mean age: 9.5 mo, range: 5-12 mo) | Anthropometric  LAZ, WAZ, WHZ  Microbial translocation:  LPS-specific IgA  LPS-specific IgG  FLA-specific IgA  FLA-specific IgG | Anthropometric  None of the biomarkers were significantly associated with risk of stunting in either the unadjusted or adjusted models  Hazard ratios of being underweight for those with concentrations of biomarker in the highest quartile compared to those in the lowest quartile:   - anti-FLA IgA: 2.02 (95% CI: 1.11, 3.67; P-trend = 0.007), - anti-LPS IgA: 1.84 (95% CI: 1.03, 3.27; P-trend = 0.02) - anti-FLA IgG: 1.94 (95% CI: 1.04, 3.62; P-trend = 0.009) - anti-LPS IgG: 2.31 (95% CI: 1.25, 4.27; P-trend = 0.01)   Microbial translocation:  Mean concentrations of anti-FLA IgA, anti-FLA IgG, anti-LPS IgA, and anti-LPS IgG at 12 mo were significantly higher than those of healthy Boston children at 9.5 mo  Anti-FLA IgG, anti-LPS IgA, and anti-LPS IgG were also significantly higher at 6 mo compared to healthy Boston babies at 9.5 mo | There was a clear trend of increasing anti-LPS and anti-FLA IgA and IgG concentrations over the first year of life.  An increased concentration of each biomarker was associated with a significant increase in risk of underweight at 6 wk, but no association with stunting present.  Limitation: The study was ubable to quantify the amount of IgG at 6 wk of age that crossed the placenta from the mother compared with that which was produced by the infant | Definition: subclinical condition of the small intestine that is characterized by villous atrophy, crypt hyperplasia, increased intestinal permeability, inflammatory cell infiltrate, and malabsorption |
| 2016  **Naylor C et al**  The impact of environmental enteropathy and systemic inflammation on infant growth failure  To examine the relationship between intestinal and systemic inflammation biomarkers with poor growth in the first two years of life. | Urban Bangladesh  (same population as the 2015 PROVIDE study)  Infants 0 – 18 mo | Longitudinal  n=700 | Anthropometric  HAZ  Intestinal inflammation:  Reg1B  MPO  Calprotectin  Systemic inflammation:  CRP  Ferritin  sCD14 | Reg1B, MPO, and calprotectin, measured in the stool at 12 weeks of age, negatively correlated with growth  CRP, ferritin, and sCD14 strongly negatively correlated with growth  Maternal health had strong positive correlations on growth.  A model containing Reg1b, MPO, ferritin, sCD14, CRP, maternal education, and maternal height explain 46.3% of variance observed in ∆HAZ  Cluster analysis yielded three distinct clusters that lead to poor growth:   - Cluster 1 = systemic inflammatory markers, including diarrhoeal burden - Cluster 2 = enteric inflammatory markers, as well as CRP and sCD14. - Cluster 3 = maternal health and SES status markers. | Systemic inflammation, intestinal inflammation, and maternal health may all be independent factors that lead to poor growth  Both systemic and intestinal inflammation had a negative impact on linear growth | *Abstract only*  Experimental Biology 2016 Meeting; abstract published in The FASEB Journal.  Definition: subclinical inflammation of the intestinal tract  No biomarker cutoffs provided |
| 2016  **Ordiz M et al**  Environmental Enteric Dysfunction is Associated with Poor Linear Growth and Can be Identified by Host Fecal mRNAs  To determine whether a single L:M test predicts subsequent linear growth in these rural African children and the extent to which fecal host mRNAs predict L:M | Rural Malawi  Children 12-61 mo  Exclusion:  - severe malnutrition  - diarrhoea within the last 3 days  - congenital abnormalities  - chronic debilitating illness | Longitudinal cohort  n=798 | Anthropometry  HAZ, WHZ  Permeability:  4-h L:M sugar absorption test  -- 5g L: 1g M  Transcriptomics | Anthropometry  n=140 (18%) no EED  n=524 (66%) moderate EED  n=134 (17%) severe EED  L:M was inversely correlated with ∆HAZ (r=0.32, P<0.001) and severe EED was associated with ∆HAZ in the subsequent 3 months (P<0.0001).  Transcriptomics  18 host mRNA transcripts were associated with L:M; 7 were identified as predictors of EED:   - CDX1, HLA-DRA, MUC12, REG1A, S100A8, TNF (sensitivity = 84%, specificity = 73%) - TNF, HLA-DRA, MUC12, CD53 (sensitivity = 84%, specificity = 83%) | L:M was a significant predictor of ∆HAZ  Severe EED can be predicted by a small number of host fecal mRNAs using random forest modeling with high sensitivity and specificity  Systemic immune response: TNF, HLA-DRA  Epithelial barrier function: MUC12  Limitations:  - study population age did not extend to 0-11 mo | No definition of EED provided  EE (without dysfunction part) defined as increased systemic inflammation in an asymptomatic child living in an unsanitary environment  EED Cutoff  *none: L:M < 0.15 moderate: 0.15< L:M< 0.45*  *severe: L:M > 0.45.* |
| 2016  **Syed S et al**  Serum anti-flagellin and anti-lipopolysaccharide immunoglobulins as predictors of growth in Pakistani infants at risk for environmental enteric dysfunction  To assess the associations between anti-FLA and anti-LPS antibodies in children at risk of EE | Rural Pakistan  Children 0-18 mo | Prospective cohort  n = 380 | Anthropometric  HAZ, WAZ, WHZ  Translocation and permeability  Serum anti-FLA- and anti-LPS-specific IgA  Serum anti-FLA- and anti-LPS-specific IgG | At 6 months:   - higher anti-LPS IgA levels were associated with a decrease in HAZ scores over 18 months (beta = -0.29, 95% CI: -0.05 to -0.54, P = 0.009).   At 9 months:   - higher anti-FLA IgA levels were associated with a decrease in HAZ scores over 18 months (beta = -0.29, 95% CI: -0.04 to -0.55, P = 0.04) - higher anti-FLA IgG levels were associated with a decrease in HAZ scores over 18 months (beta = -0.27, 95% CI: -0.01 to -0.53, P = 0.07)   anti-LPS IgG and anti-FLA IgG were not associated with ∆HAZ  Biomarkers were not associated with WAZ or WHZ scores, except for anti-LPS IgG at 6 months, with increase in WHZ over 18 months (beta = 0.26, 95% CI: -0.04 to 0.56, P = 0.01) | EED, as measured by anti-FLA IgA and anti-LPS IgA antibodies at 6 and 9 months, is associated with declines in HAZ.  Serum levels of anti-FLA-IgA in early infancy are associated with stunting. | *Conference abstract only*  Definition: an acquired condition of the small intestine that is linked to poor growth |
| 2016  **Semba R et al**  Metabolic alterations in children with environmental enteric dysfunction  Report the relationship between increased intestinal permeability and specific serum metabolites | Southern Rural Malawi  12-59 mo  Excluded: children with evidence of kwashiorkor, congenital or chronic disease, caretaker-reported diarrhea, or were under treatment for malnutrition. | Cross-sectional  n=315  n=68 no EED  n=247 EED  (L:M ratio ≥ 0.15) | Permeability:  L:M  Serum metabolites:  The 139 metabolites analyzed included 22 amino acids, 3 biogenic amines, 6 amino acid metabolites, 15 sphingolipids, 8 acylcarnitines, and 85 glycerophospholipids.  Systemic inflammation  KTR | 14 serum metabolites were significantly related with gut permeability  Negatively correlated with L:M:  - tryptophan  - ornithine  - citrulline  - four lysophosphatidylcholines  (lysoPC a C16:0, lysoPC a C18:0, lysoPC a C18:2, lysoPC a C20:4)  - two acyl-alkyl-phosphatidylcholines (PC ae C40:1, PC ae C44:3)  -two sphingomyelins (SM C16:0, SM C16:1)  Positively correlated with L:M:  - glutamate  - serotonin  - taurine  Adj Spearman correlations between gut permeability and the serotonin/tryptophan: 0.27 (P = 7.34 × 10^−7^) and kynurenine/tryptophan ratio: 0.25 (P = 6.44 × 10^−6^) | Authors suggests several serum metabolites as candidate biomarkers for EED: citrulline, ornithine,glutamate, taurine, serotonin, tryptophan, serotonin/tryptophan ratio, kynurenine/tryptophan ratio, and phos- phatidylcholines and sphingomyelins.  However, the correlations between specific serum metabolites and gut permeability were modest, which may limit their use as biomarkers. | Definition: villous shortening, crypt hyperplasia, and lymphocytic infiltration  Cutoff  *EED: L:M ≥ 0.15* |
| 2016  **Uddin M et al**  Biomarkers of Environmental Enteropathy are Positively Associated with Immune Responses to an Oral Cholera Vaccine in Bangladeshi Children  To determine how host factors impact immune responses in children receiving oral cholera vaccine. | Urban Bangladesh  Children 3-14 yo  Excluded:  healthy, no diarrhea in preceding 14 days; HAZ score less than -2 | Case-crossover  n = 40 total  n = 20, young children (3-5 yo, mean = 4.8)  n = 20 older children (7-14 yo, mean = 10.5) | Anthropometric  HAZ  Microbial translocation  EndoCAb IgG  LPS-specific IgA, IgG, IgM  Intestinal Inflammation  MPO  AAT  i-FABP  Systemic Inflammation  sCD14 | Microbial ranslocation  LPS positively associated with:   - vitamin D (p = 0.02) - i-FABP (p = 0.01) - sCD14 (p = 0.07)   Intestinal Inflammation  MPO positively associated with:   - anti-LPS IgA (p = 0.02) - CTB IgG (p = 0.03)   Systemic Inflammation  sCD14 higher in older children | Contrary to previous studies, EED, as evidenced by enteropathy biomarkers, was positively associated with increased immunogenicity to vaccine antigens | Definition: acquired syndrome, characterized by villous blunting, crypt hyperplasia, and increased intraepithelial lymphocytes and pro- inflammatory cytokine responses |
| 2016  **Yu J et al**  Environmental Enteric Dysfunction Includes a Broad Spectrum of Inflammatory Responses and Epithelial Repair Processes  To determine the association between %L and expression levels of protein coding genes (transcriptome) | Malawi  12-61 month olds residing in 1 or 6 communities in rural Malawi  Excluded: Children with chronic disability or disease, severe acute malnutrition, or receiving therapy for tuberculosis | Prospective Cohort  n=259  n=60  No EED  (%L < 0.2)  n= 157  Intermediate EED  (0.2 < %L < 0.7)  n=42  Severe EED  (%L > 0.7) | Anthropometric  HAZ, WAZ, MUACZ  Permeability:  4-h dual sugar absorption test, 1g M: 5g L  %L  Transcriptomics  51 host RNA transcripts of the human gastrointestinal tract | Permeability  %L was associated with reduced linear growth (smaller ∆HAZ) in the subsequent 3-month period (*P* < .01)  51 transcripts were correlated significantly to %L  18 most common transcripts with EED (p-values for both Pearson and Spearman < .01)  ACSL1  AQP9*  BCL2A1  CD53  CSF3R  IFI30  IL1RN  LAPTM5  LCP1  LYN*  LYZ*  MNDA* *(Pearson p = 0.012)*  PIK3AP1  PLEK*  SELL*  SLC2A3  SORL1  TAGAP  *also associated with ∆HAZ after normalization | Almost all of the 51 transcripts code for immunologically active proteins, such as IgG or IgE, or for cytokines that modulate the immune response; these are made in response to parasites, bacteria, and viruses  A relatively high proportion of the 18 most common transcripts showing a correlation with EED are associated with granulocyte colony-stimulating factor (G-CSF), which aids in differentiating dendritic cells and mobilizing neutrophils | Definition:  Cutoff  No EED: %L < 0.2  Intermediate:  0.02 < %L < 0.7  Severe: L% > 0.7 |
| 2015  **Benzoni N et al**  Plasma endotoxin core antibody concentration and linear growth are unrelated in rural Malawian children aged 2-5 years.  To examine the association of serum EndoCab versus linear growth and lactulose-mannitol assay results | Rural Malawi  Children 2-5 yo  (same study population as Weisz 2012)  Excluded: children with any chronic debilitating illness, including known HIV infection or obvious congenital abnormalities, evidence of acute malnutrition, and a recent history or current case of diarrhea or hematochezia. | cross-sectional  n=388 total analyzed for L:M  n=48 no EED  n=340 EED | Anthropometric  MUACZ, WAZ, HAZ  Permeability:  %L, %M, L:M  Microbial translocation:  log-EndoCab | Anthropometric  n = 301 (78%) stunted  No significant association between log-EndoCab and HAZ (B = −0.078, P = 0.14) or change in HAZ (B = −0.018, P = 0.27)  Permeability  n = 340 (88%) with EED, as determined by L:M ratio  No significant association between log-EndoCAb and log-%L (B < 0.001, P = 0.98) or log-L:M (B = 0.021, P = 0.62). | EndoCAb was not associated with linear growth nor with measurements of permeability and is therefore not a sensitive biomarker for these measurements | Definition: subclinical condition with T-cell infiltration of the duodenum and jejunum, decreased villous height and increased crypt depth  Cutoff  L:M > .10 is abnormal |
| 2015  **Etheredge A et al**  Markers of environmental enteric dysfunction (EED) are associated with neurodevelopment in Tanzanian children  To evaluate biomarkers of gastrointestinal epithelial and barrier function associated with EED as predictors of neurodevelopment | Tanzania  Children 6wk | Longitudinal cohort  n = 107 | Enterocyte mass  citrulline  Microbial translocation and systemic immune response  LPS-specific IgA  LPS-specific IgG  FLA-specific IgA  FLA-specific IgG  Neurodevelop-mental testing  Bayley Scales of Infant Development-III | At 6wk, negative association between LPS-IgA and expressive language score (p = 0.03)  At 6 months, positive association between expressive language association score and FLA-IgG (p = 0.02) and citrulline (p = 0.02)  At 12 months, negative association between citrulline and expressive language score (p = 0.04) | Higher levels of LPS-IgA at 6 weeks and citrulline at 12 months, and lower levels of FLA-IgG and citrulline at 6 months were associated with below-median language scores | No definition of EED provided |
| 2015  **George CM et al**  Geophagy is associated with environmental enteropathy and impaired growth in children in rural Bangladesh  To determine the relationship between geophagy, EED, and stunting | Rural Bangladesh  Children 6-30 mo (median age: 17mo)  Exclusion: no chickens present in compound | Longitudinal cohort  n = 324 eligible  (37% LTFU)  n = 205 included in 9-month follow-up | Anthropometric  HAZ, WAZ, WHZ  Intestinal Inflammation  AAT, MPO, NEO: combined to form EE disease activity score  Calprotectin  Observed and caregiver-reported geophagy | Anthropometric  At 9 months, 34% had low HAZ, 27% had low WAZ, and 7% had low WHZ  Odds of stunting for those with observed geophagy was 1.81 (95% CI: 0.85, 3.89) times that of those without observed geophagy  Odds of stunting for those with caregiver-reported geophagy was 2.27 (95% CI: 1.14, 4.51) times that of those without caregiver-reported geophagy  No significant association between observed or caregiver-observed geophagy and WAZ or WHZ  Intestinal Inflammation  Significant association (p < 0.05) between calprotectin and AAT; calprotectin and MPO; AAT and MPO  No significant association between baseline fecal markers of EED (calprotectin and EED activity score) in the highest versus lowest quartile and stunting or wasting  Significant association between being underweight and:   - EED scores in the highest versus lowest quartile (OR: 3.73, 95% CI: 1.38, 10.12) - AAT concentrations in the highest versus lowest quartile (OR: 3.17, 95%CI: 1.19, 8.43) | Significant association between caregiver-reported geophagy in the past week and elevated EE disease activity scores and calprotectin  No significant associations found betweengeophagy observed during the 5-hour structured observation period and EE markers and stunting  - This may be explained by short observation periods | Definition: disorder defined by abnormal intestinal morphology, reduced intestinal barrier function, and increased inflammation |
| 2015  **Gosselin K et al**  Serum citrulline does not predict stunting or environmental enteric dysfunction in Tanzanian and Malawian infants  To examine the relationship between serum citrulline and L:M, and citrulline and stunting at 18 mo | Tanzania and Malawi  Tanzania  Children 6wk – 18 mo  Malawi  Children 1-3 yo | cohort  Tanzania  n = 413  Malawi  n = 102 | Permeability:  L:M  Mucosal mass:  Citrulline | Tanzania  Mean (SD) citrulline level at 6 wk = 18.0 (5.8) μmol/L.  No significant difference in hazard for children in lowest quartile of citrulline compared to other quartiles (HR = 1.07, 95%CI: 0.64, 1.78)  Malawi  No significant difference in mean citrulline levels between stunted and non-stunted children (p = 0.60)  No association between L:M and citrulline (p = 0.65) or HAZ and citrulline (p = 0.31) | Citrulline did not predict subsequent stunting in Tanzania or correlate with markers of EED in Malawi. | *Abstract only*  No definition of EED provided |
| 2015  **Lima AA et al**  Risk factors, gut function biomarkers and growth deficit associated with environmental enteropathy and malnutrition: The case-control MAL-ED study in Fortaleza, Ceara, Brazil  To evaluate the variables related to the child and the mother, as well as environmental and socio-economic factors associated with environmental enteropathy and malnutrition | Brazil site within the MAL-ED study  Children 6-24 mo  Inclusion: WAZ = -1 | Case-control  n = 402 enrolled  n = 244 preliminary analysis  (126 controls, 118 cases) | Anthropometric  HAZ, WAZ  Intestinal inflammation  MPO  AAT  Calprotectin | Intestinal Inflammation  Calprotectin was significantly higher in malnourished versus nourished children  Higher MPO and AAT were associated with impaired "catch-up" growth | Calprotectin, MPO, and AAT were all associated with gut inflammation and impaired growth | *Abstract only*  No definition of EED provided |
| 2015  **Naylor C et al**  Environmental Enteropathy, Oral Vaccine Failure and Growth Faltering in Infants in Bangladesh  (PROVIDE study)  To examine the association between EED and impaired performance of the oral polio vaccine (OPV) and rotavirus vaccine | Urban Bangladesh  Children 0-2 yo (recruited at birth and followed for 2 years) | Longitudinal cohort  n = 700 | Anthropometric  HAZ, WAZ, WHZ  Intestinal inflammation  MPO  NEO  Calprotectin  AAT  REG1B  Systemic inflammation  cytokines  CRP  ferritin  sCD14 | Cluster analysis yielded three distinct clusters that lead to poor growth:   - Cluster 1 = systemic inflammatory markers, including diarrhoeal burden - Cluster 2 = enteric inflammatory markers, as well as CRP and sCD14 and two of the sanitation markers (absence of an open sewer and access to a toilet/septic tank) - Cluster 3 = maternal health, HAZ at 18 weeks, and SES status markers, including income, access to treated water, and no shared toilet.   Based on cluster analysis, REG1B, calprotectin,NEO, MPO, and AAT were considered to be biomarkers of EED  Anthropometric  REG1B, MPO both negatively associated with ∆HAZ and ∆WAZ from enrolment to age 1 yo  ∆HAZ and ∆WAZ were associated with retinol binding protein, vitamin D, zinc, and ferritin  Intestinal inflammation  Significant associations:   - OPV2—REG1B - OPV3—REG1B, calprotectin - Rotarix IgA—AAT - Rotarix success—REG1B, NEO   Systemic inflammation   - ferritin—OPV2, tetanus, pertussis, diphtheria, ∆WAZ, ∆HAZ - IL-10—Rotarix IgA - activin—diphtheria - IL-1b—∆WAZ - CRP—pertussis, ∆WAZ - sCD14—OPV1, measles, tetanus, Rotarix® success, ΔHAZ - CRP—∆HAZ | EE was associated with failure of Rotarix® and underperformance of OPV and predicted the development of malnutrition  EE and systemic inflammation biomarkers had negative associations with growth, indicating that localized enteric inflammatory damage and broad systemic inflammation both are likely to have an important effect on nutritional status | Definition: subclinical illness characterized by small intestine inflammation with shortened villi, intestinal barrier dysfunction, and reduced nutrient absorption |
| 2014  **Jones KD et al**  Mesalazine in the initial management of severely acutely malnourished children with environmental enteric dysfunction: A pilot randomized controlled trial  To investigate the safety and acceptability of mesalazine as a treatment for children with severe acute malnutrition (SAM) | Urban Kenya  Children 12-60 mo with SAM and stunting  Exclusion:  HIV, TB, bloody diarrhea, evidence of renal or hepatic impairment, thrombocytopenia or severe anemia, if they were receiving treatment from another facility, if they had medical difficulties in normal feeding, pre-existing renal disease, asthma, hypersensitivity or salicylates, or if they were on medication known to interfere with the action of the study drug | Double-blind RCT  n = 44  (22 mesalazine, 22 placebo)  Mesalazine arm: 20/22 completed follow-up  (all 22 included in final analysis)  Placebo arm: 19/22 completed follow-up  (all 22 included in final analysis) | Intestinal inflammation  Calprotectin  Microbial translocation  Anti-EndoCAb IgG  Systemic inflammation  IFNα  IFN-γ  sCD14  TNFβ  IL-1α, IL-1RA, IL-7, IL-8, IL-10, IL-15, IL-17a, IL-22, IL-31  Growth hormone axis  IGF-1 | Intestinal inflammation  Baseline levels of fecal calprotectin were higher than has been reported from healthy control populations in high-income countries and sub-Saharan Africa  Anthropometric  Significantly greater MUAC growth in the placebo arm to day 56  No differences in HAZ at any time (baseline, day 28, day 56) between mesalazine and placebo groups  Microbial translocation  IGF-1 and EndoCAb were positively correlated with each other and negatively correlated with linear growth throughout the study (p = 0.01 and p = 0.04, Figure 3c) | Pharmacologically-mediated reduction in EED is well tolerated, providing the first clear evidence that EED is likely to be at least partly maladaptive  Because edema can alter MUAC and most of the children in this study were edematous at enrolment, the clinical significance is unclear  Mesalazine was safe to use and did not have any adverse effects, but also does not appear to significantly improve the outcomes of SAM or EED | Definition: acquired syndrome of reduced intestinal barrier and absorptive function, characterised by histopathological changes of small intestinal villous atrophy and crypt hyperplasia, accompanied by lymphocytic infiltration of the lamina propria  SAM defined as MUAC <11.5 cm or bilateral pedal edema  EED was inferred on the basis of stunting and chronic inflammation (erythrocyte sedimentation rate (ESR) >20 mm/hour) |
| 2014  **Prendergast A et al**  Stunting Is Characterized by Chronic Inflammation in Zimbabwean Infants  To examine the relationship between the growth hormone-IGF axis and stunting as well as numerous biomarkers of EED | Zimbabwe  HIV-negative mother-infant pairs (Infants 0 mo followed to 24 mo)  Exclusion: infant weight < 1500 g, acutely life-threatening conditions, mother planned to leave city after delivery | Case-control  n = 202 total (101 cases, 101 controls) | Anthropometric  HAZ, WAZ, WHZ, MUAC  Microbial translocation  sCD14, IgG EndoCAb  Intestinal damage  i-FABP  Systemic Inflammation  CRP, AGP, IL-6  Growth hormone axis  IGF-1, IGFBP3 | Anthropometric  Cases had significantly lower HAZ and WAZ than controls at each time-point between birth and 18 mo  In adjusted models,   - higher levels of IGF-1 between 6 wk – 12 mo were associated with reduced odds of stunting at 18 mo (p < 0.001) - higher log_10_ levels of CRP and AGP between 6 wk – 12 mo were associated with increased odds of stunting (p = 0.008, p = 0.087)   There were no associations between levels of I-FABP, IL-6, sCD14 or EndoCAb and stunting  Growth hormone-IGF axis  IGF-1 and IGFBP3 levels were similar for cases and controls at birth, but after 6 wk both protein levels were significantly lower in cases than controls  Inflammation versus IGF-1   - At birth, IGF-1 was strongly associated with inflammation (AGP: p < 0.001, CRP: p < 0.001, and sCD14: p = 0.031) - Negative associations were found at every subsequent time-point to 12 mo of age, but by 18 mo associations were no longer present   Microbial translocation  sCD14 was higher in controls at birth and again at 18 mo, but levels in the time in between were similar between cases and controls  IgG EndoCAb levels were similar between groups throughout follow-up  Inflammation  IL-6 highly elevated in cases at birth, declined over first 6 wk, then rose again after 6 wk  CRP and AGP increased from 6 wk and were higher in cases after 12 mo; however, by 18 mo they were not significantly higher (and in some instances, control levels were higher than case levels)  *Analysis of mother versus infant levels*   - Mothers of cases had lower levels of IGF-1 (221.4, 95% CI: 239.8, 23.1) - WAZ at birth was associated with maternal IGF-1 (R = 0.21, p = 0.003) and infant IGF-1 (R = 0.51, p < 0.0001) - HAZ at birth was not associated with maternal IGF-1 and was weakly associated with infant IGF-1 (R = 0.23, p = 0.046) - Maternal IGF-1 associated with maternal weight (p = 0.029) and height (p=0.013) - No significant association in inflammatory markers between mothers of cases and controls - Strong association between maternal and infant AGP (R = 0.52, p = 0.001), CRP (R = 0.46, P = 0.010) and sCD14 (R = 0.39, P < 0.001) - Weak association between maternal and infant IL-6 (R = 0.31, P = 0.087) | Stunting is influenced by both maternal and infant factors  Low-grade inflammation and the IGF-1 axis were associated in the first year of life (but not to 18 mo)  Lower levels of IGF-1 are likely to mediate stunting in early life  By 18 mo, levels of IGF-1 were similar between groups — this may suggest a window of opportunity in which interventions to reduce inflammation and increase IGF-1 may improve linear growth  Infant inflammation markers were closely related to maternal inflammation at birth — this may imply that inflammation during pregnancy may “set” the infant inflammatory axis, which influences the level of IGF-1 in early life  I-FABP was not significantly associated with stunting, but levels were exceptionally high in both groups compared to healthy individuals from wealthier populations  Reduction in I-FABP in both groups between 12-18 mo may suggest that the mucosa repair beyond infancy | Definition: villous blunting, inflammatory infiltrate and increased intestinal permeability  Cases: HAZ < -2 @18mo  Controls: HAZ > -0.5 @18mo |
| 2014  **Ryan KN et al**  Zinc or albendazole attenuates the progression of environmental enteropathy: A randomized controlled trial  To determine if zinc and/or albendazole improve EED, as measured by the L:M test | Rural Malawi  Asymptomatic children 1-3 yo  Exclusion: 3 or more loose stools per day, chronic debilitating illnesses such as HIV, cerebral palsy or congenital abnormalities; severe malnutrition, or children needing acute medical treatment | Double-blind 3-arm RCT  - zinc supplement  - Albendazole supplement  - Placebo  n = 234 enrolled  n = 222 analyzed  (72 zinc, 73 albendazole, 77 placebo) | Permeability  4-h dual sugar absorption test, 1g M: 5g L  L:M ratio, %L  Anthropometric  HAZ, WAZ | Permeability  95% of children in study had evidence of EE, as measured by L:M ratio  Mean baseline L:M was 0.32 – 0.18 among all children and did not differ significantly between the 3 groups  L:M ratio increased for all groups, despite interventions  ∆L:M significantly different in each intervention group compared to placebo:   - Zinc: 0.03 ± 0.20 (p < 0.03) - Albendazole: 0.04 ± 0.22 (p < .04)   Anthropometric  No significant differences in weight gain or lineargrowth among the 3 groups  No results reflecting ∆HAZ and L:M | Both zinc and albendazole interventions stopped the progression of EE as measured by L:M ratio  Neither intervention had an effect on anthropometric measures; however, no long-term growth was measured | Definition: subclinical condition manifested by T-cell infiltration of the small-bowel mucosa and diffuse villous atrophy  Cutoff  Abnormal L:M > 0.30  Albendazole = anti-helminthic therapy |
| 2014  **Smith HE et al**  Multiple micronutrient supplementation transiently ameliorates environmental enteropathy in Malawian children aged 12-35 months in a randomized controlled clinical trial.  To test whether micronutrient and fish oil supplementation would ameliorate EED, as measured by the L:M test | Rural Malawi  Children 12-35 mo  Exclusion: bloody diarrhea, inability to provide 4-h urine collection, chronic or debilitating illness, SAM, the first twin of a pair (second received same therapy but was not recorded) | Double-blind 3-arm RCT  A. MN/FO  B. MN/placebo  C. Plcbo/plcbo  A. n = 80 enrolled, 1 LTFU, 78 L:M after 12 wk, 79 L:M after 24 wk  B. n = 80 enrolled, 2 LTFU, 78 L:M after 12 wk, 76 L:M after 24 wk  C. n = 75, enrolled, 2 LTFU, 72 L:M after 12 wk, 73 L:M after 24 wk | Permeability  4-h dual sugar absorption test, 1g M: 5g L  L:M ratio, %L  Anthropometric  linear growth, weight gain | Permeability  No results about L:M vs HAZ  All children had abnormal L:M values at baseline   - Group A: 0.44 (0.39, 0.50) - Group B: 0.49 (0.41, 0.56) - Group C: 0.43 (0.38, 0.49)   The two intervention groups had significant improvements L:M after 12 wk compared to baseline:   - Group A: 0.35 (0.30, 0.40) - Group B: 0.37 (0.32, 0.43) - Group C: 0.38 (0.33, 0.44)   All groups had significant improvement in L:M after 24 wk compared to the baseline:   - Group A: 0.35 (0.30, 0.40) - Group B: 0.38 (0.33, 0.43) - Group C: 0.37 (0.30, 0.44)   L:M ratio from 12-24 wk   - Group A did not change - Group B increased by 0.01 unit - Group C decreased by 0.01 unit   Anthropometric  Significant weight gain in Group A (MN/FO) versus Group C (placebo) (p = 0.01)  No significant change in linear growth between intervention and placebo groups | No significant difference in L:M between the intervention group and the placebo group at 12 wk (P = 0.06)  All groups had similar L:M ratios after 24 wk, suggesting the interventions were transient and limited  The improvement in L:M in the C group after 24 wk suggeststhat factors other than nutrient intake may play an important role in EE. | Definition: characterized by diffuse small bowel villous atrophy with T cell infiltration  Cutoff  Clinically significant = ∆L:M > .06 units |
| 2013  **Agapova et al**  Detection of low-concentration host mRNA transcripts in Malawian children at risk for environmental enteropathy  To develop a method for detecting human mRNA suggestive of EE in feces | Rural Malawi  Children ages 2-5 yo  Exclusion: no diarrhea in the last 7 days, and none of the children was known to be infected with HIV, nor had a chronic congenital condition | Case control  n = 70  34 cases, with EED  36 controls, without EED | Permeability and absorption  dual-sugar absorption  1 g M: 5 g L  L:M ratio  Transcriptomics | Transcriptomics  Top six potential markers of EED:  - REG4 best differentiated children with increased L:M from children with normal L:M (p = 0.01)  - TJP1, LAP3, REG1B, IL-22, and BDEF1 were not significantly different between children with and without EED  Genes for MPO, calprotectin, TNF, IFNg, and other markers of systemic inflammation (interleukins) were not significantly different between children with and without EED | Human mRNA that is present in extremely low copynumbers can consistently and reproducibly be isolated and detected in a set of stool samples. | Definition - diffuse villous atrophy of the small bowel associated with inflammatory T-cellinfiltration, of unknown aetiology  Cutoff  no EED:  L:M < 0.10  (Note: comparisons were made between children with L:M > 0.24 and L:M < 0.13) |
| 2013  **Kosek M et al**  Fecal markers of intestinal inflammation and permeability associated with the subsequent acquisition of linear growth deficits in infants.  To clarify the ability of established markers of intestinal inflammation and permeability to predict linear growth | Pilot study for MAL-ED (8 sites: BGD, INV, NEB, PKN, BRF, PEL, SAV, TZH)  *MAL-ED = Malnutrition and Enteric Diseases  Children at birth, followed to 9 mo  Exclusion: Stool samples from children with diarrhea in last 7 days or L:M testing on the day of/before stool collection were omitted | Prospective cohort  n = 661 children  (744 stool samples for which all three biomarkers were available) | Anthropometric  LAZ  Intestinal Inflammation  AAT, MPO, NEO: combined to form EE disease activity score | Anthropometric  MPO, NEO, and AAT all predicted declines in LAZ in the 6 mo following the test  A child with a max EED score (10) would be expected to lose 0.47 LAZ scores more than a child with the lowest score (0) in the six months following the test   - p < 0.01 in Brazil and Nepal - p < 0.10 in South Africa   Intestinal Inflammation  All three tests predicted declines in LAZ in the 6 months following the test.  Correlations between MPO, NEO, and AAT were low (all below 0.22) | Levels of intestinal inflammation are associated with subsequent acquired deficits in linear growth in infants over 6 months  Composite score of three biomarkers was made possible by the low correlation between the three markers  -- This allowed accounting for a greater degree in growth deficits than can be explained by any marker by itself  Using stool markers is markedly cheaper/more convenient than L:M urine testing | Definition: increased crypt depth, decrease in villus height, and lymphocytic infiltration |
| 2013  **Lin A et al**  Household Environmental Conditions Are Associated with Enteropathy and Impaired Growth in Rural Bangladesh  To assess the relationship between fecal environment contamination and EED, parasite burden, and growth | Rural Bangladesh  0-48 mo (mean age at collection time: 35 mo)  Excluded: children with diarrhea, vomiting, or a perigenital skin infection on the day of the L:M test (n=3) | Cohort  n = 136 in sample  (17 LTFU)  n=119 total  (66 clean environment, 53 contaminated environments) | Anthropometric:  HAZ, WAZ, WHZ  Permeability:  5-h urine collection  250mg/mL L, 50mg/mL M  L:M ratio  Systemic Inflammation:  Total IgG  Microbial translocation  EndoCab IgG  Parasitic infection  *Ascaris*  *Trichuris*  *Giardia*  *Cryptosporidium*  *Entamoeba*  hookworm | Mean L:M ratio:   - clean = 0.21 - contaminated = 0.31   *Children from clean households (compared to contaminated households):*  Anthropometric   - L:M strongly associated with HAZ (beta = –0.33, 95% CI: −0.62, −0.05) and WAZ scores (−0.24, 95% CI: −0.47, −0.01) - None of the anthropometric measurements were strongly associated with IgG EndoCAb or total IgG titers - 0.54 SDs (95% CI: 0.06, 1.01) higher HAZ - 22% lower number of stunted individuals (95% CI: -2%, -42%) - no other outcomes (WAZ, WHZ, HCZ, proportion wasted/underweight) were significantly associated after adjustment   Permeability   - 0.32 SDs (95%CI: −0.72, 0.08) lower L:M ratio in urine   Microbial translocation   - 0.24 SDs (95% CI: −0.63, 0.16) lower IgG EndoCAb titers - 0.54 SDs (95% CI: −0.95, 0.13) lower IgG EndoCAb titers, after adjusting for potential confounders   Parasitic infection  Children living in clean household environments had lower prevalence of all detectable parasites measured compared with children in contaminated household environments, but none of them were significant after adjustment | Children in clean environments had higher HAZs and lower IgG EndoCAb titers than children living in contaminated households (after adjustment)  HAZ and WAZ strongly correlated with L:M ratio  Both clean and contaminated households had higher mean L:M ratios (0.21, 0.31) than healthy infants in UK  Even when children from clean households were surrounded by other households that did not meet our definition of a clean environment, these children still had dramatically lower stunting prevalence, lower levels of parasitic infection, and better gut function.  Limitations:  Tests and growth measured at mean 35 months; does not take into account growth from birth | Definition: disorder featuring a smallbowel with abnormal morphology and physiology, including crypt hyperplasia, villous atrophy, lymphocyte infiltration into the lamina propria and epithelium, reduced mucosal surface area, and increased intestinal permeability.    *“Contaminated environment” = a household with poor water quality (median E. coli > 10CFU/ 100mL), inadequate sanitation (open defecation, open pit latrines, slabs with broken water seals, toilets that flush to somewhere else, or hanging toilets), and unhygienic handwashing conditions (a dedicated location that lacked either water or soap or the absence of a dedicated location to wash hands) |
| 2013  **Peterson KM et al**  REG1B as a predictor of childhood stunting in Bangladesh and Peru.  To determine whether there is anassociation between stool REG1B concentrations with subsequent childhood growth deficit | Bangladesh and Peru  Children 0-24 mo (followed from birth) | Prospective cohort  n = 222 Bangl.  n = 97 Peru | Anthropometric  LAZ, WAZ, WHZ  Intestinal tissue repair  REG1B | Anthropometric  LAZ at 3 mo was significantly associated with LAZ at each subsequent month  Intestinal repair  Inflated REG1B concentrations at 3 mo were significantly associated with lower LAZ at 9^+^, 12, 15^++^, 18, 21, and 24 mo  ^+^Bengali cohort only  ^++^Peru cohort only  Higher REG1B concentrations at 3 mo were associated with future LAZ measures through 24 mo in Bengali (p = 0.006) and Peruvian (p = 0.059) cohorts  Inflated REG1B concentrations at 3 mo were not associated with lower WHZ at any month | Higher REG1B concentrations at 3 mo were significantly associated with future LAZs in two birth cohorts | No definition of EED provided; used stunting as primary indicator of EED |
| 2013  **van der Merwe L et al**  Long-chain PUFA supplementation in rural African infants: a randomized controlled trial of effects on gut integrity, growth, and cognitive development.  To assess whether early n–3 LC-PUFA supplementation improves infant intestinal integrity, growth, and cognitive function | The Gambia, rural  Children 3 mo, followed until 9 mo  Exclusion:  Severe congenital abnormalities that could affect growth/development, infants from multiple births, HIV infection | Double-blind RCT  n = 183  (92 receive treatment, 91 control)  8 (4%) LTFU  (3 treatment, 5 control)  n = 172 analyzed at 9 mo  (87 treatment, 85 control)  n = 155 attended follow-up at 12 mo  (79 treatment, 76 control) | Anthropometric  Left-side triceps, biceps, and sub-scapular skinfold thickness; MUAC; head circumference  Permeability  5-h dual-sugar absorption test  4g L : 1g M  L:M ratio  Intestinal inflammation  Calprotectin  Systemic inflammation  CRP  AGP  albumin  Plasma fatty acid status  plasma total lipids  Cognitive development  2-step means-end problem-solving test  single-object task attention assessment | Plasma fatty acid status  FO supplementation resulted in significant increases in DHA and EPA  (This was just to measure that the individuals were actually absorbing the PUFAs)  Anthropometric (ITT analysis)   - FO supplementation significantly associated with increase in MUAC (p = 0.017), tricep skinfold-thickness-for-age (p = 0.048) - No significant association between FO supplementation and linear growth (95% CI: 20.27, 0.90; P = 0.084)   Permeability   - Only 5% of infants had normal L:M ratio - No significant difference in L:M ratio between groups   Systemic inflammation  No significant association between FO supplementation and systemic inflammation markers  Intestinal inflammation   - More than 85% of infants had calprotectin concentrations higher than healthy European references - No significant association between FO supplementation and intestinal inflammation markers   Cognitive development  No significant difference in performance between treatment groups | The PUFA intervention successfully increased infant n–3 plasma FA status after 5 mo of intervention  PO supplementation may cause increase in MUAC and 3 skinfold-thickness measurements  PO supplementation not associated with any other anthropometric measurements  FO supplement did not lead to reduced degrees of intestinal and systemic inflammation or reduced rates of morbidity | Definition: characterized by intestinal villous atrophy, crypt hyperplasia, and inflammatory cell invasion of the lamina propria  EPA and DHA are types of omega-3 fatty acids  Cutoff  Normal L:M < 0.07 |
| 2013  **Wessells KR et al**  Associations between intestinal mucosal function and changes in plasma zinc concentration following zinc supplementation  To examine the relationship between intestinal mucosal function (as measured by L:M RATIO and plasma citrulline concentrations) and changes in zinc concentration following zinc supplementation | Rural Burkina Faso  Children age 6–23 mo, currently breast-feeding  Exclusion:  Hemoglobin < 60 g/L, fever, diarrhea (>3 liquid or semi-liquid stools in a 24 h period) reported in the past week, currently consuming vitamin or mineral supplements or zinc- fortified infant formulas, or demonstrate bipedal or other serious med conditions | 3-armed partially masked RCT  n = 451 in original study  3 arms:  1. 5mg Zn tablet  2. 5mg liquid Zn  3. Liquid placebo  n=282 successful completion of L:M test  n=80 subgroup analysis of children with CRP concentrations < 10mg/L | Anthropometry  LAZ, WAZ, WLZ  Permeability  2-h dual-sugar absorption test  4g L : 1g M  L:M recovery ratio (not concentration ratio)  - Note: children were allowed to eat after 1h of consuming L:M  Also looked at L:C and M:C ratios  Mucosal mass  Citrulline  Systemic inflammation  AGP  CRP  HRP*  Mineral uptake  zinc | Mucosal mass  Citrulline positively associated with age (p = 0.02)  Baseline citrulline not associated with L:M recovery ratio  ∆citrulline was positively associated with WAZ over the three weeks  Permeability  No consistent associations between urinary L:M recovery ratios at baseline and age or anthropometric status (HAZ)  Systemic inflammation  AGP and CRP were not associated with L:M  Mineral uptake  ∆PZC** was significantly greater among children who received Zn supplementation (p < 0.0001)  Negatively related to ∆PZC:   - Initial plasma Zn concentration - Child age - Child HAZ - ∆WAZ - Final AGP > 1 g/L - HRP2 > 0.75 ng/mL - Time of day   Positively related to ∆PZC:   - elapsed time since last breast-feed - baseline AGP > 1   Controlling for above factors, baseline L:M recovery ratio was negatively associated with ∆PZC, regardless of liquid or solid supplementation (p = 0.014)  No association between ∆PZC and L:M concentration ratio | Short-term Zn supplementation significantly increased PZC after 3 wk  Children with higher L:M recovery ratio at baseline had significantly smaller ∆PZC  - this trend was consistent between placebo and treatment groups (i.e. zinc supplementation will increase PZC in children with or without altered permeability)  No association between L:M and citrulline, suggesting that mucosal mass may not necessarily correlate with permeability  No association between ∆PZC and baseline Citrulline/∆Citrulline | Definition:  chronic condition of unknown etiology, characterized by changes in small bowel morphology, including villous atrophy, hyperplasia of crypt cells and infiltration of the lamina propria by inflammatory cells  Cutoff  No EED:   - L:M recovery ratio < 0.03 - L:M concentration ratio < 0.12   Alternate cutoff:   - L:M recovery ratio > 0.07 - L:M concentration ratio > 0.28   Abnormal citrulline concentration < 14 µmol/L |
| 2012  **Weisz AJ et al**  Abnormal gut integrity is associated with reduced linear growth in rural Malawian children  To determine the relationship between EED (as measured by the L:M test) to linear growth faltering | Rural Malawi  Children 2-5, only twins  Exclusion:  Chronic debilitating illness, (e.g. HIV, congenital abnormalities), evidence of acute malnutrition, recent history of diarrhea or hematochezia | Case-cohort  n = 418 | Anthropometric  HAZ, WAZ, MUACZ  Permeability  4-h dual-sugar absorption test  5g L : 1g M  L:M ratio, %L | Anthropometric  ∆HAZ associated with two %L (p < 0.05):   - %L < 0.13 - %L > 0.37   ∆HAZ not associated with L:M ratio  MLR found that initial %L, WAZ, initial HAZ, number of bicycles in home, presence of animals sleeping in home, time per day clean water was brought to home, and previous treatment for malnutrition were all significant factors in predicting linear growth  Permeability  364/418 (87%) of children had abnormal L:M ratio | This study has differing results from previous studies, which show that L:M RATIO *is* associated and %L is *not* associated  - Authors mention that “differences in the pattern of the perturbation of the mannitol and lactulose excretion and L:M between different populations may represent different insults to the children’s gastrointestinal mucosa mediated by nutritional status, age, or environment” | Definition: asymptomatic, diffuse villous atrophy of the small bowel associated with T-cell inflammatory infiltration and reduced intestinal integrity, leading to reduced intestinal nutrient absorption  Cutoff  Abnormal: L:M > .10 |
| 2010  **Kelly P et al**  Gastric and intestinal barrier impairment in tropical enteropathy and HIV: Limited impact of micronutrient supplementation during a randomised controlled trial  To examine the effect of micronutrient supplementation on gut pH (study 1) and barrier function (study 2) | Urban Zambia  Adults with or without HIV | Case-cohort (within clustered RCT)  Study 1:  n = 203, participants who had been taking MM or placebo for 4-9 mo  Study 2:  n = 86, participants taken over the course of the 4-9 mo, some before and some after crossover (for logistic reasons) | Permeability  5-h four-sugar absorption test  (0.5g xylose, 1g rhamnose, 5g lactulose, 0.2g 3-O-methyl D-glucose)  Xylose recovery, RG ratio, LR ratio  Microbial translocation  LPS  EndoCAb IgG/IgM  Systemic inflammation  TNFR (receptor p55)  Gastric pH | Gastric pH  No difference in median pH or number of individuals with hypochlorhydria between MM and placebo groups  Permeability  No difference in any of the measures of permeability between MM and placebo, nor between HIV groups  Log-transformed xylose recovery negatively correlated with log-transformed anti-LPS IgG (p = 0.006)  Microbial Translocation (n = 87)  No difference in LPS concentrations nor anti-LPS IgG between MM and placebo  Significant reduction of IgM in MM group compared to placebo  Systemic inflammation  Correlation between log- transformed TNFRp55 and anti-LPS IgM was significant (r = 0.30, P = 0.006) – more strongly correlated than IgG  Correlation between TNFRp55 and anti-LPS IgG (r = 0.27, P = 0.01) | Little evidence to support the use of MM supplementation to reduce markers of intestinal permeability or bacterial translocation, except possibly anti-LPS IgM  Anti-LPS IgM correlated with TNF pathway activation, implying that translocation is a determinant of systemic inflammation  -- Anti-LPS IgM was reduced in MM group but TNFR was not. This may imply some sort of threshold reduction in IgM before TNF pathway is affected | No definition of EED provided  Cutoff  Hypochlorhydria: gastric pH > 4.0 |
| 2010  **Papadia C et al**  Plasma citrulline as a quantitative biomarker of HIV-associated villous atrophy in a tropical enteropathy population  To determine if serum citrulline concentration is a reliable marker of early morphological and functional changes in HIV enteropathy | Urban Zambia  Adults with tropical enteropathy (included both HIV-seronegative and seropositive, not taking ART)  Exclusion: pregnant or lactating women, individuals who had experienced diarrhea and had taken antibiotics or NSAIDS in the previous month | Case-Cohort  n = 145  (44 HIV seropositive, 101 HIV seronegative) | Anthropometric  MUACZ  Permeability  5-h four-sugar absorption test  (0.5g xylose, 1g rhamnose, 5g lactulose, 0.2g 3-O-methyl D-glucose)  Xylose recovery, RG ratio, LR ratio  Enterocyte mass  Citrulline  Morphometric  Villous compartment volume (VCV)  Epithelial surface area (ESA)  Villous height (VH)  Crypt depth (CD)  Villous width (VW) | Anthropometric  MUACZ correlated with citrulline (p = 0.04) and were significantly reduced in all those with diarrhea (p = 0.003)  Permeability  Associated with citrulline levels in both HIV positive and HIV negative patients  *Note: not significant after BH correction for multiple correlations, except for xylose absorption  Morphometric  VH-CD ratio significantly lower in HIV positive individuals  Enterocyte mass  Citrulline concentrations significantly lower in HIV positive patients | Citrulline appears to be a reliable marker of small bowel structural and functional integrity in HIV-related enteropathy  Citrulline concentration does not seem to be a sufficiently sensitive marker for rejection or viral enteritis as its valuesdecline only in more severe intestinal mucosal damage | Definition—*Tropical enteropathy*: asymptomatic villous atrophy of the small bowel; characterised by subclinical malabsorption and increased permeability; mild reduction in villous height, broadening of the villi and increased crypt depth  Cutoff   - healthy:   citrulline > 30 μmol/L   - partial villous atrophy:   10< citrulline < 30 μmol/L   - total villous atrophy:   citrulline < 10 μmol/L |

Definitions

Stunted: HAZ < –2

Underweight: WAZ < –2

Wasted: WHZ < –2

Abbreviations:

AGP, α-1-acid glycoprotein;

AAT, α-1-antitrypsin;

BAZ, BMI-for-age Z-score;

Cdn-15, claudin-15;

CRP, C-reactive protein;

DHA, docosahexaenoic acid;

EED, environmental enteric dysfunction;

EFZ, endogenous fecal zinc;

EndoCab, endotoxin core antibody;

EPA, eicosapentaenoic acid;

FAZ, fractional absorbed zinc;

FLA, flagellin;

GLP-2, glucagon-like peptide-2;

GCA, glycocholic acid;

GCDCA, glycochenodexocy-cholic acid;

GDCA, glycodeoxycholic acid;

GUDCA, glycoursodeoxycholic acid;

HAZ, height-for-age Z-score;

HEV, Hepatitis E viral infection;

HIV, Human Immunodeficiency Virus;

HPLC-PAD, high-performance liquid chromatography with pulsed amperometric detection;

HRP, histidine-rich protein;

IgA, immunoglobulin A;

IgG, immunoglobulin G;

IgM, immunoglobulin M;

IGF-1, insulin-like growth factor-1;

IGFBP3, insulin-like growth factor binding protein 3;

IFN- α, interferon alpha;

IFN-γ, interferon gamma;

IL, interleukin;

KTR, kynurenine-tryptophan ratio;

LC-MSMS, liquid chromatography-tandem mass spectrometry;

L:C, lactulose-creatinine;

L:M, lactulose-mannitol;

L:R, lactulose-rhamnose;

LPS, lipopolysaccharide;

MAL-ED, Malnutrition and Enteric Disease (study)

MPO, myeloperoxidase;

MUACZ, mid-upper arm circumference Z-score;

NEO, neopterin;

NRZ, net retained zinc;

PZC, plasma zinc concentration;

PROVIDE, Performance of Rotavirus and Oral Polio Vaccines in Developing Countries (study);

sCD14, Soluble Cluster of Differentiation 14;

TAZ, total absorbed zinc

TDCA, taurodeoxycholic acid;

TNFβ, tumor necrosis factor-β;

TMCA, tauromurocholic acid;

WAZ, weight-for-age Z-score;

WHZ, weight-for-height Z-score;

%L, percent lactulose permeability;

%M, percent mannitol absorption;

%R, percent rhamnose absorption;
